# Integration of Urine Proteomic and Metabolomic Profiling Reveals Novel Insights Into Neuroinflammation in Autism Spectrum Disorder

**DOI:** 10.3389/fpsyt.2022.780747

**Published:** 2022-05-09

**Authors:** Wenlong Liu, Liming Li, Xiaochun Xia, Xulan Zhou, Yukai Du, Zhaoqing Yin, Juan Wang

**Affiliations:** ^1^Department of Child Development and Behavior, School of Medicine, Women and Children's Hospital, Xiamen University, Xiamen, China; ^2^Department of Public Health and Medical Technology, Xiamen Medical College, Xiamen, China; ^3^Department of Maternal and Child Health, School of Public Health, Tongji Medical College, Huazhong University of Science and Technology, Wuhan, China; ^4^Division of Neonatology, The People's Hospital of Dehong Autonomous Prefecture, Mangshi, China

**Keywords:** autism spectrum disorder, proteomics, metabolomics, integrated analysis, urine, neuroinflammation

## Abstract

Autism spectrum disorder (ASD) comprises a group of neurodevelopmental disorders whose etiology and pathogenesis are not fully understood. To gain insight into the molecular basis of ASD, we performed comparative integrated proteomic and metabolomic analyses of urine samples from children diagnosed with ASD and healthy children. All 160 samples underwent proteomics analysis and 60 were analyzed by liquid chromatography-mass spectrometry to obtain metabolite profiles. We identified 77 differentially expressed proteins (DEPs; 21 downregulated and 56 upregulated) and 277 differentially expressed metabolites; 31 of the DEPs including glutathione, leukocyte antigens, glycoproteins, neural adhesion factors, and immunoglobulins, have been implicated in neuroinflammation. The proteomic analysis also revealed 8 signaling pathways that were significantly dysregulated in ASD patients; 3 of these (transendothelial leukocyte migration, antigen processing and presentation, and graft vs. host disease) were associated with the neuroimmune response. The metabolism of tryptophan, which is also related to the neuroimmune response, has been found to play a potential role in ASD. Integrated proteome and metabolome analysis showed that 6 signaling pathways were significantly enriched in ASD patients, 3 of which were correlated with impaired neuroinflammation (glutathione metabolism, metabolism of xenobiotics by cytochrome P450 and transendothelial migration of leukocyte). We also found a correlation between prostaglandin (PG) E2 levels and the inflammatory response in ASD. These results underscore the prominent role of the neuroimmune response in ASD and provide potential biomarkers that can be used for diagnosis or as targets for early intervention.

## Introduction

Autism spectrum disorder (ASD) comprises a group of neurodevelopmental disorders clinically characterized by deficits in social interaction and communication, and stereotyped behaviors (1). The prevalence of ASD in the United States increased from 0.67% in 2000 to 1.85% in 2016 (2); in China, the prevalence is 7 in 1,000 (3). The etiology of ASD involves a combination of genetic, epigenetic, and environmental factors and is not fully understood.

Immune system dysregulation is a risk factor for the development and progression of ASD (4, 5), as evidenced by an aberrant cytokine response, abnormal numbers of immune cells, neuroinflammation, dysfunction of the adaptive and innate immune systems, and altered immunoglobulin levels in individuals with ASD. The pro-inflammatory factor Osteopontin (OPN) was found to be upregulated in ASD patients, which was significantly correlated with autism severity (6). In addition, children with ASD may present with several distinct immune subphenotypes that are correlated with the degree of behavioral impairment (7). Maternal immune activation—especially involving interleukin (IL)-17—was found to be a major contributor to neurodevelopmental abnormalities in a mouse model of ASD (8). These findings suggest a close association between neuroinflammation and ASD pathogenesis. On the other hand, the behavioral symptoms of children with ASD were shown to improve with fever (9), implying that inflammation has a beneficial role in ASD. Thus, the relationship between neuroinflammation and ASD remains to be elucidated.

High-throughput -omics technologies have provided molecular-level insights into normal physiology as well as into disease states, including ASD. Proteomic analysis revealed 41 proteins related to endoplasmic reticulum stress, unfolded protein response, acute-phase response, inflammatory response, and endocytosis with altered levels in peripheral blood mononuclear cells of patients with ASD compared with the control subjects (10). Using a proteomic approach, another study identified 2 brain regions with dysregulated glutamate receptor signaling, glutathione (GSH)-mediated detoxification, and fatty acid oxidation in children with ASD (11). Metabolomic studies have shown that the levels of metabolites associated with abnormal amino acid metabolism, oxidative stress, and gut microbiome were altered in children with ASD (12, 13). It was recently reported that 3 metabolic pathways (pyrimidine, ubiquinone, and vitamin K metabolism) were perturbed in children with ASD, with a panel of central nervous system biomarkers (9-hexadecenoylcarnitine [C16:1] and phosphatidylcholine [PC ae-C36:1]) found by metabolomic analysis (14). Integrated analyses of -omics data have identified potential biomarkers and revealed their interrelationships (15–18). However, to date, there have been no studies that have applied a combination of metabolomic and proteomic approaches to the investigation of ASD etiology.

In this study, we performed comparative proteomic and metabolic profiling of urine samples from children with ASD and normal children in order to investigate the contribution of neuroinflammatory mechanisms to ASD pathogenesis. Our results provide insight into the molecular basis of ASD and biomarkers that can support diagnosis or serve as targets for therapeutic interventions.

## Materials and Methods

### Ethics Approval

This study was conducted in accordance with the 1965 Declaration of Helsinki and its later amendments. The study protocol was approved by the Ethics Committee of Tongji Medical College, Huazhong University of Science and Technology (Hubei, China) (approval no: IORG0003571). Written, informed consent was obtained from the parents of participants.

### Study Participants and Sample Collection

A total of 160 Chinese children including 80 with ASD and 80 with typical development (TD), were enrolled in the study. Children with ASD (age range: 2–7 years old) without other systematic diseases or neurodevelopmental disorders were recruited from Xiamen Women and Children's Hospital, Xiamen, Fujian, China and evaluated by a neuropsychiatrist specializing in autism. The diagnosis of ASD was made based on the Diagnostic and Statistical Manual of Mental Disorders, 5th Edition (19). Gender- and age-matched children with TD were recruited from schools in Xiamen.

Midstream urine samples (50 ml) were collected in sterile tubes in the morning and immediately placed on dry ice to prevent bacterial growth. After thawing for 30 min at room temperature, the samples were centrifuged at 3,000 × *g* for 15 min at 4°C and stored at−80°C until use.

### Capillary Flow Data-Independent Acquisition–Based Proteomic Analysis

A 5-ml urine sample was obtained from each participant and 10 samples were combined for proteomic analysis. A total of 16 pooled samples (8 each from patients with ASD and subjects with TD) were analyzed.

DIA proteomic analysis was carried out as described previously (20–22). High-pH reverse-phase separation was conducted using a liquid-phase system (LC-20AB; Shimadzu, Tokyo, Japan). The elution peak was monitored at 214 nm and one component was collected every minute. The sample was combined with the elution peak of the chromatogram to obtain 10 components and then freeze-dried.

Dried peptide samples were reconstituted and centrifuged and then separated using an ultrahigh-pressure liquid chromatography (UPLC) system (Ultimate 3000; Thermo Fisher Scientific, Waltham, MA, USA). Peptides were separated with mobile phase A (2% acetonitrile+0.1% formic acid) and mobile phase B (98% acetonitrile+0.1% formic acid) at a flow rate of 500 nl/min using the following program: 5% phase B for the first 5 min, phase B (5–35%) from 5 to 160 min, phase B (35–80%) from 160 to 170 min, 80% phase B from 170 to 175 min, and 5% phase B in the final 5 min. The peptides separated by the liquid phase were sprayed through a nano-electrospray ionization source into a tandem mass spectrometer (Q Exactive HF; Thermo Fisher Scientific) for data-dependent acquisition (DDA) and DIA analyses.

For DIA on the Q Exactive HF system, the detection parameters were set at a resolution of 30,000, automatic gain control target of 1e5, the scan range of 350–1,500 m/z, loop count of 50, automatic maximum inject time, and filter dynamic exclusion duration of 30 s. DDA data were identified using MaxQuant v1.5.3.30. Spectronaut™ v11.0 (Biognosys, Zurich, Switzerland) was used to construct a spectral library. Peptide/protein entries with a false discovery rate (FDR) ≤ 1% were used to construct the final spectral library. Data were reviewed based on the UniProtKB/SwissProt *Homo sapiens* proteome database. The R package MSstats (www.bioconductor.org/packages/release/bioc/html/MSstats.html) was used to screen differentially expressed proteins (DEPs) according to a fold change ≥ 2 or ≤ 0.83 and *p* < 0.05.

To identify the signaling networks altered in ASD, the DEPs identified in the present study were subjected to functional analysis using Database for Annotation, Visualization, and Integrated Discovery (david.ncifcrf.gov/). The Gene Ontology (GO) and Kyoto Encyclopedia of Genes and Genomes (KEGG) databases were used to search for categories and pathways.

### Metabolite Profiling

A total of 60 urine samples (30 individual ASD samples and TD samples) were used for metabolic analysis, which was performed as described previously (23–25). Urine samples were slowly thawed at 4°C, and 100 μl of the sample was combined with 300 μl of precooled methanol and mixed by vortexing for 1 min. The mixture was stored at −20°C for 2 h and then centrifuged at 4,000 × *g* at 4°C for 30 min. A 250-μl aliquot of supernatant from each sample was set aside for liquid chromatography-tandem mass spectrometry (LC-MS/MS) analysis. A quality control (QC) sample was prepared by mixing equal volumes of each sample.

A UPLC system (2777C; Waters, Milford, MA, USA) equipped with an Acquity UPLC HSS T3 column (100 × 2.1 mm, 1.8 μm; Waters) was used to separate urine metabolites. Mobile phase A was water and 0.1% formic acid, while mobile phase B was acetonitrile and 0.1% formic acid. The gradient elution program was as follows: 0–1 min, 99% A; 1–3 min, 85–99% A; 3–6 min, 50–85% A; 6–9 min, 50–95% A; 9–10 min, 5% A; and 10–12 min, 1% A. The injection volume for each sample was 10 μl, and the flow rate was set to 0.4 ml/min.

A high-resolution tandem mass spectrometer (Xevo G2 XS QTOF; Waters) was used to detect metabolites. Samples were analyzed in positive and negative ion modes. The capillary voltage and sampling cone voltage were set to 3.0 kV and 40.0 V, respectively, in positive ion mode and to 2.0 kV and 40.0 V, respectively, in negative ion mode. The time-of-flight mass range was from 50 to 1,200 Da and the scan time was 0.2 s. For MS/MS detection, all the precursors were fragmented using 20–40 eV with a scan time of 0.2 s. To evaluate the stability of the LC-MS/MS system during acquisition, a QC sample was obtained every 10 samples.

For metabolomic analysis, raw data files were created using Progenesis QI v2.2 software (Waters) for peak alignment and selection. Data were normalized with the projected quasi-Newton method (26), and low-quality ions with relative standard deviation (RSD) > 30% were filtered out. *p*-values were adjusted with the Benjamini–Hochberg method for multiple hypothesis testing, and the FDR was limited to 0.05.

Metabolomic data were mean-centered, pareto-scaled, and analyzed by principal component analysis (PCA) and partial least squares discriminant analysis (PLS-DA). The quality of the models was evaluated with the relevant R^2^ and Q^2^ values (27). The variable influence in projection (VIP) value in the PLS-DA model was calculated to determine its contribution to the classification. Metabolites with VIP > 1.0 were evaluated with the Student's *t*-test at the univariate level to determine the significance of each metabolite. A corrected *P*-value (*q* value) < 0.05 was considered statistically significant. Log2 fold change was used to determine how the selected metabolites differed between the ASD and TD groups.

The Human Metabolome Database and KEGG database were used to confirm candidate differentially expressed metabolites (DEMs). Pathway analyses of DEMs were performed with MetaboAnalyst v5.0 (www.metaboanalyst.ca) (28).

### Integrated Proteomic and Metabolomic Data Analyses

MetaboAnalyst v5.0 was used to perform integrated pathway analyses of DEPs and DEMs (29). To integrate the pathway data, DEPs and DEMs were simultaneously projected into pathways in the KEGG database.

### Statistical Analyses

Statistical analyses were carried out using SPSS v19.0 (IBM, Armonk, NY, USA). Data are reported as the mean ± SD. Normally distributed data were analyzed with the Student's *t*-test to assess the significance of differences between the ASD and TD groups. A *p* < 0.05 was considered statistically significant.

## Results

### Demographics of the Study Population

The study population comprised 80 children with ASD and 80 with TD. The demographic characteristics of the participants are presented in Table 1. There was no significant difference in age or sex ratio between groups (*P* > 0.05).

**Table 1:** Demographic information of ASD group and TD group.

**Characteristics**		**Groups**		***P*-value**
		**TD(*n* = 80)**	**ASD(*n* = 80)**	
Age^a^ (means ± SD, years)		4.51 ± 1.03	4.73 ± 1.21	0.234
Gender^b^	Male	53	64	0.074
	Female	27	16	

*TD, typically developing group; ASD, autism spectrum disorders; SD, Standard Deviation*.

a *Analyzed using the Student's t-test*.

b *Analyzed using the Chi-squared test*.

### Proteomic Profiling

**Figure 1:**
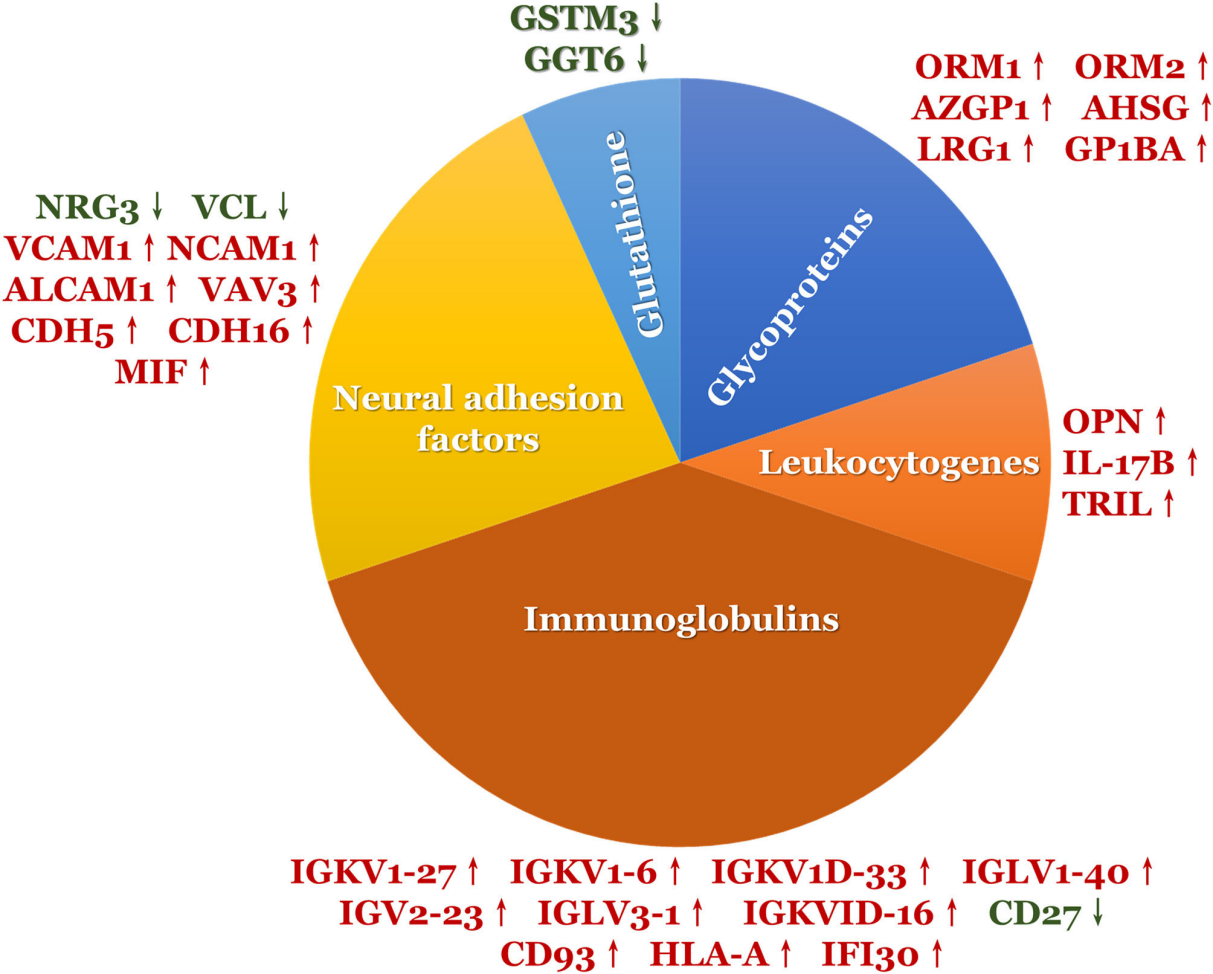
The DEPs involved in neuroinflammation.

A total of 2,700 proteins were identified using a DIA-based proteomic method. There were 77 DEPs (56 upregulated and 21 downregulated) between the ASD and TD groups (Table 2 and Supplementary Figure 1). Of these, 31 (40%) were associated with neuroinflammation (Figure 1), including 26 that were upregulated and 5 that were downregulated in the ASD group compared with the TD group. Immunoglobulins accounted for the largest proportion of these proteins; the remaining proteins were neural adhesion factors, glycoproteins, leukocyte antigens, and GSH.

**Table 2:** List of differentially expressed proteins.

**Asscession no**.	**Protein descriptive**	**Abbreviation**	**Log2FC**	***p*-Value**
P10451	Osteopontin isoform OPN-a precphosursor	OPN	1.238▲	0.038
Q9UHF5	Interleukin-17B isoform 1 precursor	IL17B	1.027▲	0.005
Q7L0X0	TLR4 interactor with leucine rich repeats precursor	TRIL	1.052▲	0.024
A0A075B6S5	Immunoglobulin kappa variable 1–27	IGKV1-27	1.128▲	0.049
A0A0C4DH72	Immunoglobulin kappa variable 1–6	IGKV1-6	1.234▲	0.034
P01593	Immunoglobulin kappa variable 1–33	IGKV1D-33	1.017▲	0.013
P01703	Immunoglobulin lambda variable 1–40	IGLV1-40	2.112▲	0.024
P01705	Immunoglobulin lambda-chain, partial	IGLV2-23	1.156▲	0.040
P01715	Immunoglobulin lambda variable 3–1	IGLV3-1	1.451▲	0.015
P01601	Immunoglobulin variable region VK-NHL104	IGKV1D-16	1.103▲	0.043
Q9NPY3	Complement component C1q receptor	CD93	1.023▲	0.023
P10314	HLA class I histocompatibility antigen, A-32 alpha chain	HLA-A	1.168▲	0.012
P13284	Gamma-interferon inducible lysosomal thiol reductase	IFI30	1.138▲	0.037
P19320	Vascular cell adhesion protein 1 isoform a precursor	VCAM1	1.437▲	0.003
P13591	Neural cell adhesion molecule 1 isoform 2 precursor	NCAM1	2.087▲	0.008
Q13740	CD166 antigen isoform 1 precursor	ALCAM1	1.004▲	0.005
Q9UKW4	Guanine nucleotide exchange factor VAV3 isoform 1	VAV3	3.628▲	0.035
O75309	Cadherin-16 isoform 1 precursor	CDH16	1.132▲	0.031
P33151	Cadherin-5, type 2 preproprotein variant, partial	CDH5	2.146▲	0.003
P14174	Macrophage migration inhibitory factor	MIF	1.427▲	0.008
P02763	Alpha-1-acid glycoprotein 1	ORM1	1.397▲	0.016
P19652	Alpha-1-acid glycoprotein 2 precursor	ORM2	1.325▲	0.011
P25311	Zinc-alpha-2-glycoprotein precursor	AZGP1	1.139▲	0.007
P02765	Alpha-2-HS-glycoprotein isoform 2 preproprotein	AHSG	1.111▲	0.011
P02750	Leucine-rich alpha-2-glycoprotein precursor	LRG1	1.396▲	0.046
P07359	Platelet glycoprotein Ib alpha chain precursor	GP1BA	1.338▲	0.016
O00468	Agrin	AGRN	1.005▲	0.015
P51170	Amiloride-sensitive sodium channel subunit gamma	SCNN1G	1.641▲	0.012
P35318	ADM precursor	ADM	1.305▲	0.025
P61769	Beta-2-microglobulin	B2M	1.017▲	0.010
P06396	Gelsolin	GSN	1.046▲	0.011
P01258	Calcitonin isoform CT preproprotein	CALCA	1.066▲	0.015
P10645	Chromogranin-A isoform 1 preproprotein	CHGA	1.632▲	0.032
Q9BY43	Chromatin-modifying protein 4a	CHMP4A	1.026▲	0.038
Q9H6B4	CXADR-like membrane protein	CLMP	1.030▲	0.019
Q96PD2	Discoidin, CUB and LCCL domain-containing protein 2 precursor	DCBLD2	1.194▲	0.013
P35555	Fibrillin	FBN	1.983▲	0.031
Q08830	Fibrinogen-like protein 1 precursor	FGL1	1.753▲	0.028
P07093	Glia-derived nexin isoform a precursor	SERPINE2	1.194▲	0.031
P16520	Guanine nucleotide-binding protein G(I)/G(S)/G(T) subunit beta-3 isoform 1	GNB3	1.618▲	0.037
P24593	Insulin-like growth factor-binding protein 5 precursor	IGFBP5	1.601▲	0.010
Q96MU8	Kremen protein 1	KREMEN1	1.181▲	0.049
Q9Y5K2	Kallikrein-4	KLK4	1.413▲	0.009
Q6UX15	Layilin isoform 1 precursor	LAYN	1.015▲	0.013
Q13449	Limbic system-associated membrane protein isoform 1 preproprotein	LSAMP	1.048▲	0.043
Q08397	Lysyloxidase homolog 1 preproprotein	LOXL1	1.532▲	0.013
Q16819	Meprin A subunit alpha precursor	MEP1A	1.151▲	0.020
P15586	N-acetylglucosamine-6-sulfatase precursor	GNS	1.012▲	0.009
P34059	N-acetylgalactosamine-6-sulfatase isoform 1 precursor	GALNS	1.322▲	0.012
Q6UXB8	Peptidase inhibitor 16 precursor	PI16	1.420▲	0.009
Q96S96	Phosphatidylethanolamine-binding protein 4	PEBP4	1.043▲	0.031
Q13103	Secreted phosphoprotein 24 precursor	SPP2	1.043▲	0.033
Q9NY72	Sodium channel subunit beta-3 precursor	SCN3B	1.159▲	0.003
Q9Y5X3	Sorting nexin-5 isoform a	SNX5	1.050▲	0.028
P11684	Uteroglobin precursor	SCGB1A1	1.973▲	0.005
Q8WY21	VPS10 domain-containing receptor SorCS1 isoform a precursor	SORCS1	1.495▲	0.046
P26842	CD27 antigen	CD27	−1.758▼	0.043
P56975	Pro-neuregulin-3, membrane-bound isoform isoform X10	NRG3	−3.155▼	0.022
P18206	Vinculin isoform meta-VCL	VCL	−1.421▼	0.009
P21266	Glutathione S-transferase Mu 3	GSTM3	−1.132▼	0.031
Q6P531	Gamma-glutamyltransferase 6 isoform	GGT6	−1.714▼	0.016
P04745	Alpha-amylase 1	AMY1A	−1.064▼	0.020
P19961	Alpha-amylase 2B	AMY2B	−1.048▼	0.003
Q16706	Alpha-mannosidase 2	MAN2A1	−1.092▼	0.021
P54802	Alpha-N-acetylglucosaminidase precursor	NAGLU	−1.277▼	0.011
P25705	ATP synthase subunit alpha, mitochondrial isoform a precursor	ATP5F1A	−3.722▼	0.005
Q53H82	Endoribonuclease LACTB2	LACTB2	−2.413▼	0.017
P06744	Glucose-6-phosphate isomerase isoform 3	GPI	−2.230▼	0.003
O00461	Golgi integral membrane protein 4 isoform 1	GOLIM4	−1.775▼	0.015
P04746	Pancreatic alpha-amylase precursor	AMY2A	−1.084▼	0.012
Q9BTY2	Plasma alpha-L-fucosidase precursor	FUCA2	−1.698▼	0.012
Q9UQ80	Proliferation-associated protein 2G4	PA2G4	−2.631▼	0.041
Q6PHR2	Serine/threonine-protein kinase ULK3	ULK3	−1.996▼	0.031
Q13813	Spectrin alpha chain, non-erythrocytic 1 isoform X6	SPTAN1	−2.467▼	0.048
P04066	Tissue alpha-L-fucosidase precursor	FUCA1	−1.485▼	0.004
O00159	Unconventional myosin-1c	MYO1C	−1.126▼	0.043
P30050	60S ribosomal protein L12	RPL12	−3.738▼	0.046

▲ *up-regulated*; ▼ *down-regulated*.

### Protein Enrichment Analysis

**Figure 2:**
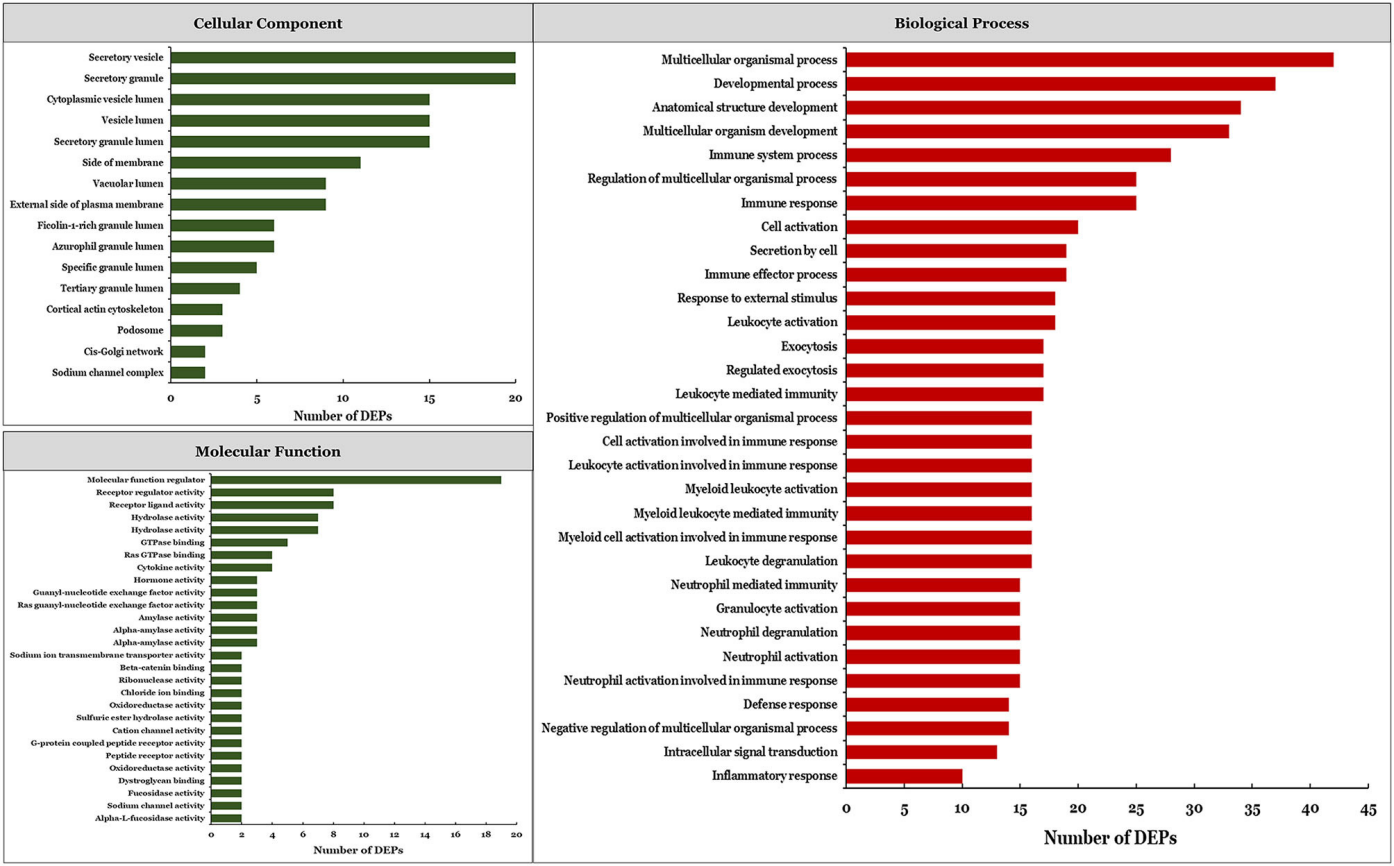
Significantly enriched GO terms based on DEPs.

We performed a functional enrichment analysis of DEPs and identified significantly enriched terms (Figure 2). There were 16, 28, and 131 significant terms in the GO categories of cellular component, molecular function, and biological process, respectively (*P* < 0.05). The most enriched terms in the cellular component category were secretory vesicle and secretory granule, whereas the most enriched molecular function term was molecular function regulator. The top-ranked enriched terms in the biological process category were multicellular organism process, developmental process, anatomical structure development, multicellular organism development, and immune system process. Among the significantly enriched biological process terms, 38 were related to immune response (Supplementary Table 1) including immune system process, immune response, and immune effector process, among others.

**Figure 3:**
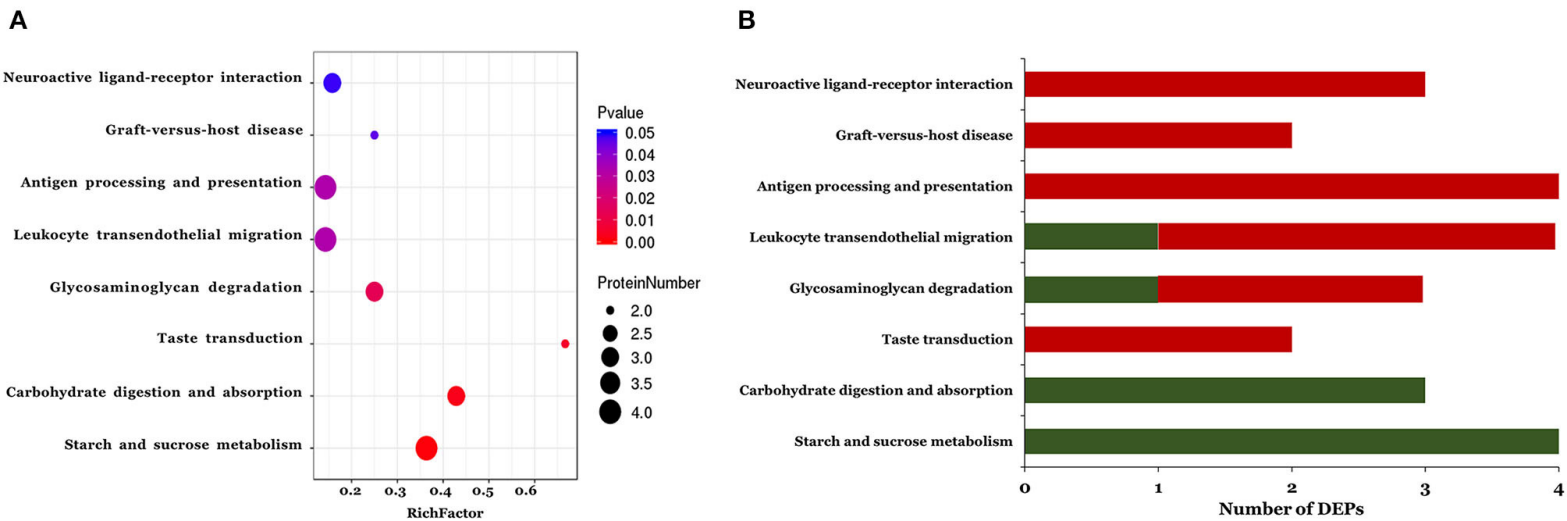
The KEGG pathway enrichment analysis of the differentially expressed proteins. A two-tailed Fisher's exact test to test the enrichment of the differentially expressed protein. **(A)** Significantly enriched KEGG pathway; **(B)** Mapped differential expressed proteins number of KEGG pathway.

The KEGG pathways associated with the DEPs identified in the ASD group are shown in Figure 3. In total, eight pathways were significantly enriched including starch and sucrose metabolism, carbohydrate digestion and absorption, taste transduction, glycosaminoglycan degradation, leukocyte transendothelial migration, antigen processing and presentation, graft-vs. -host disease, and neuroactive ligand-receptor interaction (Figure 3A and Table 3). Notably, 3 of the 8 pathways (leukocyte transendothelial migration, antigen processing and presentation, and graft-vs. -host disease) were associated with the immune response. In addition, the expression of DEPs related to starch and sucrose metabolism and carbohydrate digestion and absorption was decreased whereas that of DEPs involved in taste transduction, antigen processing and presentation, graft-vs.-host disease, and neuroactive ligand-receptor interaction was increased (Figure 3B).

**Table 3:** The significantly enriched KEGG pathways of DEPs.

**Pathway name**	**Pathway ID**	***P*-value**	**Level 1**	**Level 2**	**Proteins**
Starch and sucrose metabolism	ko00500	0.001	Metabolism	Carbohydrate metabolism	AMY1A; AMY2A; GPI; AMY2B
Carbohydrate digestion and absorption	ko04973	0.003	Organismal Systems	Digestive system	AMY1A; AMY2A; AMY2B
Taste transduction	ko04742	0.006	Organismal Systems	Sensory system	GNB3; SCNN1G
Glycosaminoglycan degradation	ko00531	0.014	Metabolism	Glycan biosynthesis and metabolism	GNS; GALNS; NAGLU
Leukocyte transendothelial migration	ko04670	0.032	Organismal Systems	Immune system	VCL; VCAM1; CDH5; VAV3
Antigen processing and presentation	ko04612	0.032	Organismal Systems	Immune system	HLA-A; IFI30; AZGP1; B2M
Graft-vs.-host disease	ko05332	0.046	Human Diseases	Immune diseases	HLA-A; AZGP1
Neuroactive ligand-receptor interaction	ko04080	0.048	Environmental Information Processing	Signaling molecules and interaction	CALCA; AHSG; ADM

### Metabolomic Analysis

**Figure 4:**
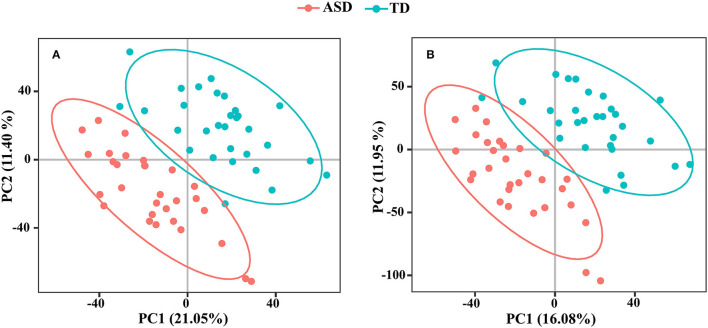
The PLS-DA score plots from ASD case (red) and TD control (green) in positive ion mode **(A)** and negative ion mode **(B)**.

We detected 7,667 and 9,837 features in positive and negative ion modes, respectively. The number of features in QC samples with RSD ≤ 30% was 6,674 in positive ion mode and 8,202 in negative ion mode (87.05 and 83.38%, respectively). The results of PCA showed that the pooled QC sample was clustered in positive and negative ion modes (Supplementary Figure 2). The PLS-DA revealed a significant difference between the ASD and TD groups (Figure 4). The reliability of the PLS-DA model was assessed by monitoring the goodness of fit (R^2^) and predictive ability (Q^2^), which were 0.811 and 0.468, respectively, in positive ion mode and 0.799 and 0.420, respectively, in negative ion mode. These results suggested that the stability and reproducibility of the models were sufficient to resolve DEMs between children with ASD and TD.

A total of 168 metabolites were differentially expressed (VIP ≥ 1.0 and *q* < 0.05) in positive ion mode (Supplementary Table 2) including 105 that were upregulated and 63 that were downregulated in the ASD group compared to the TD group. In negative ion mode, 109 metabolites were significantly altered (VIP ≥ 1.0 and *q* < 0.05) including 73 that were upregulated and 36 that were downregulated in the ASD group compared to the TD group (Supplementary Table 3).

### Metabolite Enrichment Analysis

**Figure 5:**
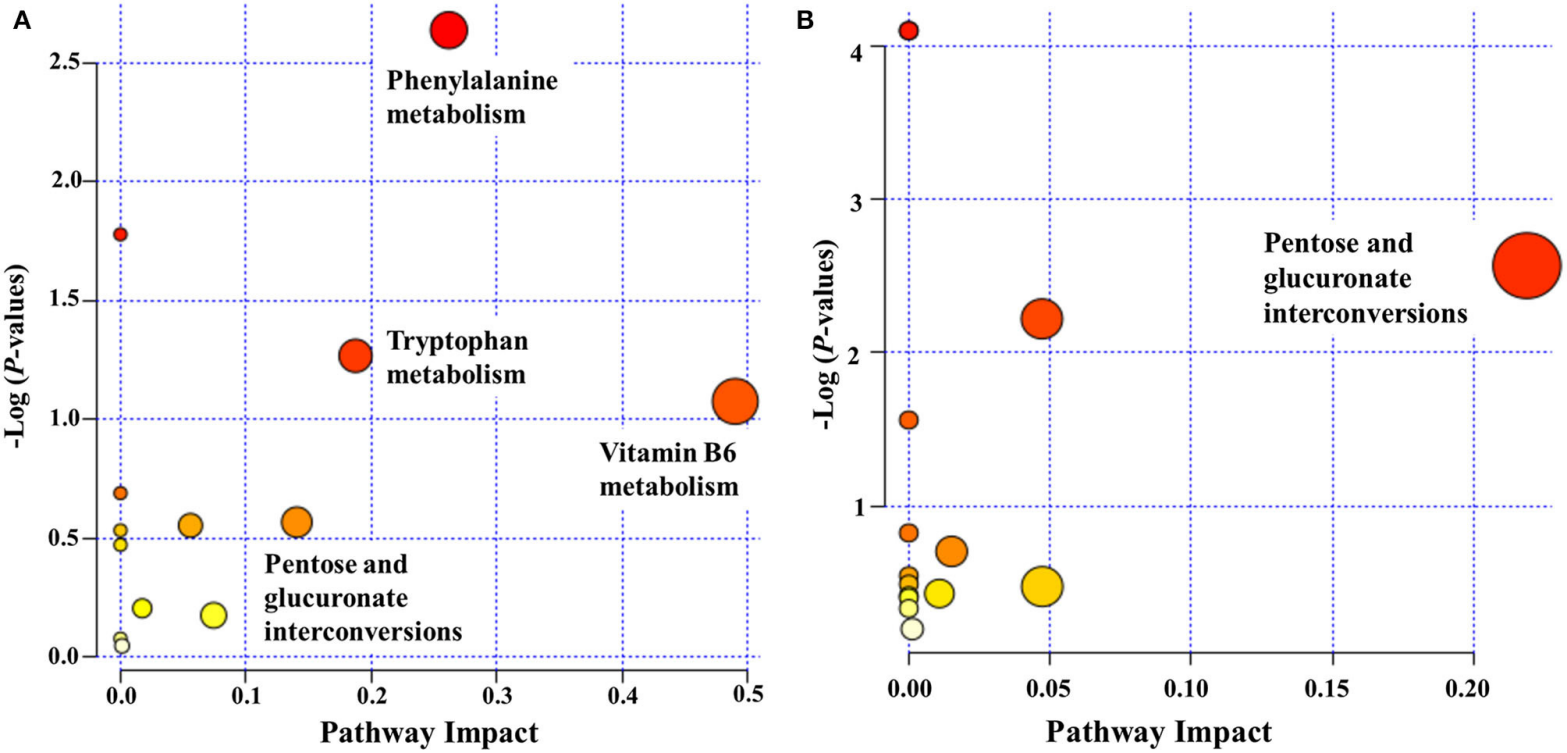
Functional classification of differentially accumulated metabolites between ASD and TD groups in positive ion mode **(A)** and negative ion mode **(B)**. The “metabolome view” shows the pathway impact on the X-axis vs. the negative log (*P*-value) on the Y-axis for the metabolic pathways. The pathways that were most significantly changed are characterized by both a high-log (P) value and a high-impact value (top right region).

We examined pathways corresponding to significantly altered metabolites in ASD using MetaboAnalyst according to FDR < 0.05 and an impact value >0.1. Four processes—namely, phenylalanine metabolism, vitamin B6 metabolism, tryptophan metabolism, and pentose and glucuronate interconversions—were markedly enriched in positive ion mode in the ASD group compared with the TD group (Figure 5A). Only 1 pathway, pentose and glucuronate interconversions, was significantly enriched in negative ion mode (Figure 5B).

### Integrated Analysis of Proteome and Metabolome Data

**Figure 6:**
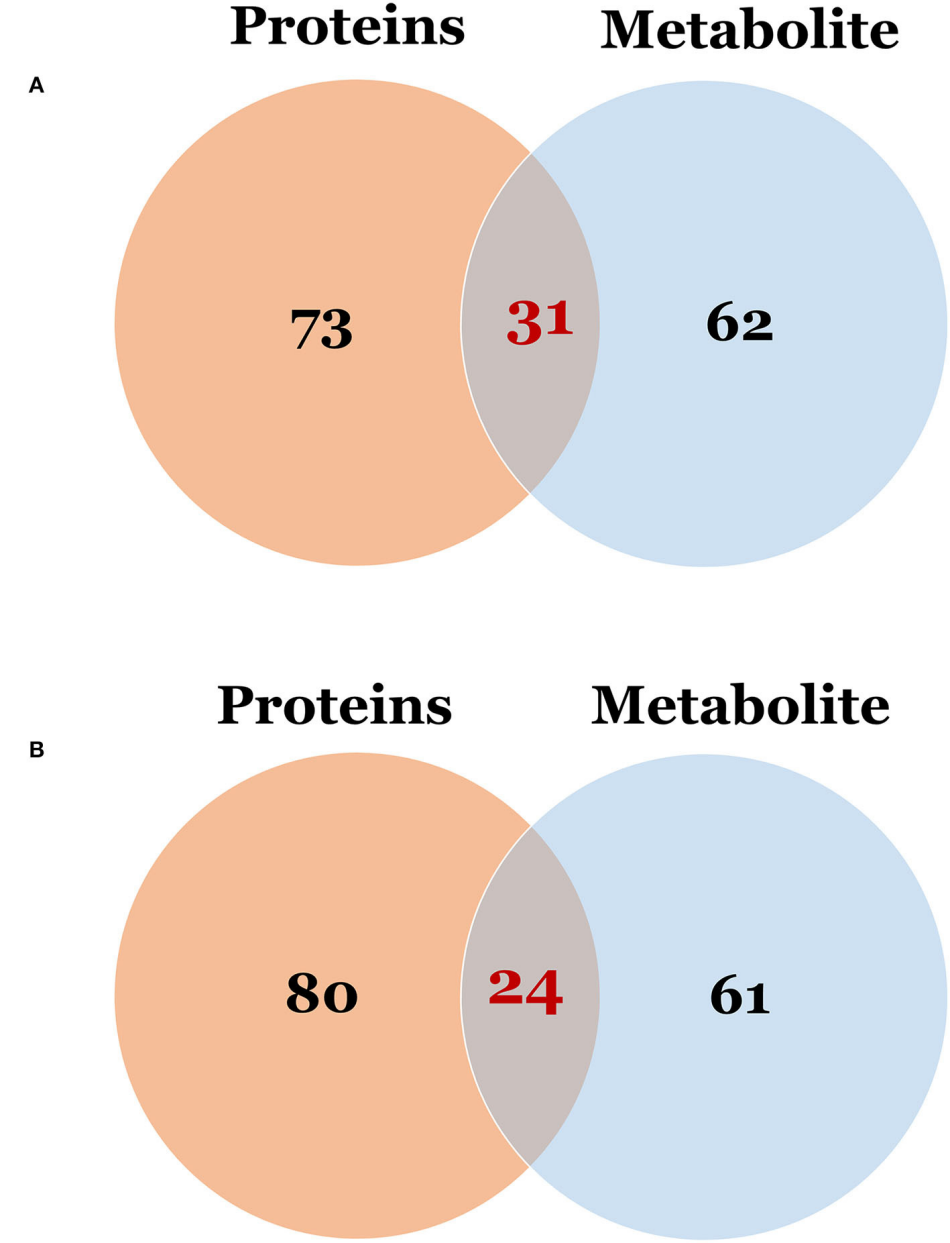
The Venn diagram of the pathway in which differentially expressed proteins and metabolites were involved. **(A)** Differentially expressed proteins vs. metabolites in positive ion mode; **(B)** Differentially expressed proteins vs. metabolites in negative ion mode.

**Figure 7:**
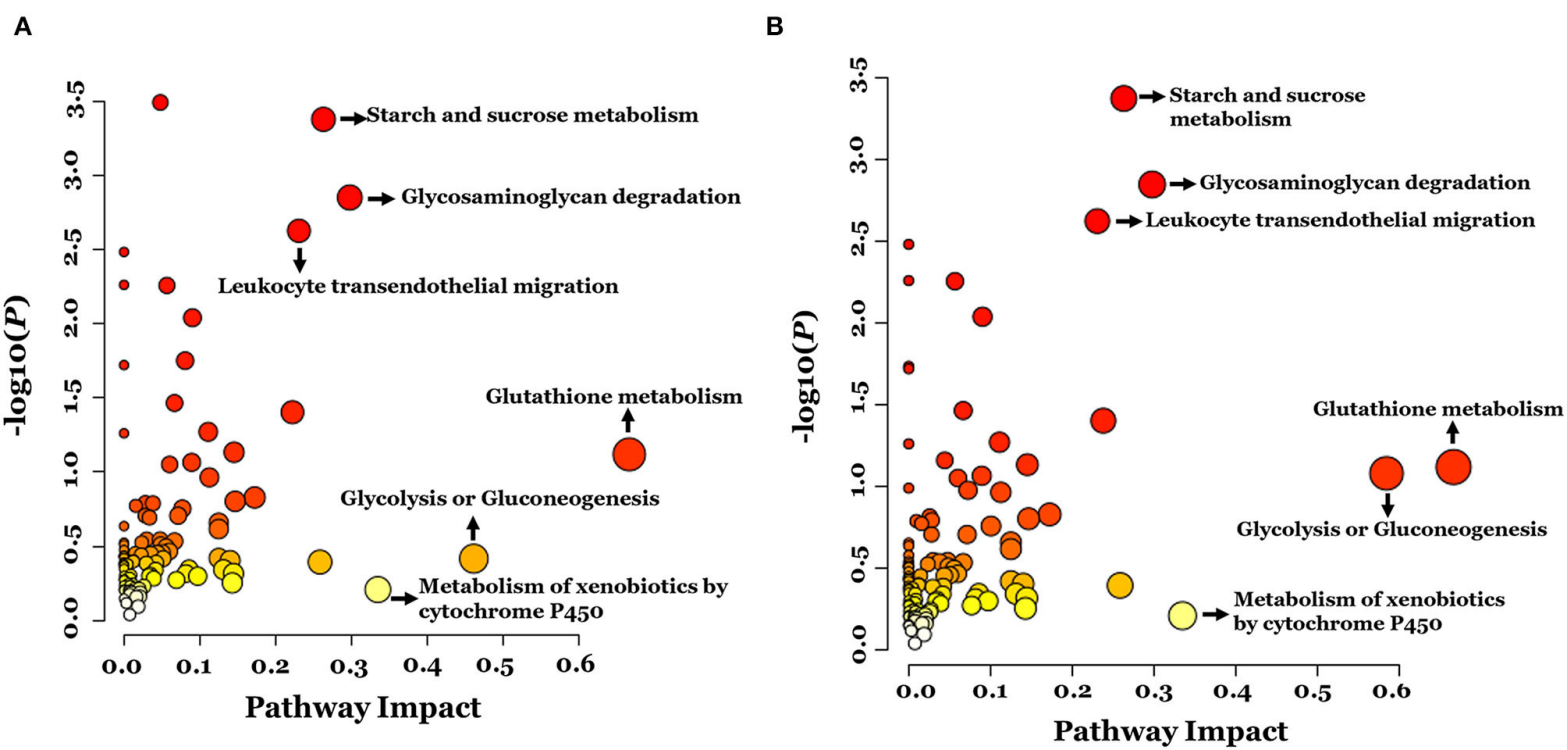
Integrated pathway analysis of proteins and metabolites differentially regulated between ASD and TD groups in positive ion mode **(A)** and negative ion mode **(B)**.

To examine global changes in protein and metabolite profiles between the ASD and TD groups, we mapped DEPs and DEMs and extracted pathways containing both proteins and metabolites using the “Joint Pathway Analysis” tool in MetaboAnalyst v5.0. The Venn diagram revealed 31 metabolic pathways in positive ion mode and 24 in negative ion mode that were associated with the DEPs and DEMs (Figure 6). Integrated proteomic and metabolomic analyses showed 6 altered pathways in the ASD group compared with the TD group including GSH metabolism, glycolysis or gluconeogenesis, metabolism of xenobiotics by cytochrome P450, glycosaminoglycan degradation, starch and sucrose metabolism, and leukocyte transendothelial migration (Figure 7). Three of these pathways were correlated with dysregulation of neuroinflammation—namely, GSH metabolism, metabolism of xenobiotics by cytochrome P450, and leukocyte transendothelial migration.

## Discussion

This study investigated the molecular basis of ASD pathogenesis with a combined proteomic and metabolomic approach. Immune dysfunction is thought to be involved in the etiology of ASD (4, 5). In this study, 40% of DEPs identified by the proteomic analysis were related to neuroinflammation and 3 associated protein pathways were related to the immune response. In the integrated proteomics and metabolomics analysis, 3 pathways were found to be correlated with dysregulation of neuroinflammation. These results provide theoretical evidence of the role of neuroinflammation in ASD pathology.

Children with ASD often exhibit abnormal redox signaling, especially in GSH metabolism (30). Synergistic and antagonistic interactions between glutamate and GSH can result in neuronal dysfunction. Chen et al. (31) revealed that 18 oxidative stress-related markers were dysregulated in the blood of children with ASD. These 18 oxidative stress markers included glutathione metabolism, transmethylation cycle and trans-sulfuration pathways, vitamins, and trace elements. In this study, the integrated analysis showed that GSH metabolism was significantly altered in ASD, which was accompanied by decreased expression of GSH S-transferase Mu 3 (GSTM3) and γ-glutamyltransferase 6 isoform (CGT6) in the ASD group. A previous study reported an imbalance in the GSH/GSH disulfide ratio and depletion of GSH in children with ASD, implying that the GSH system plays a protective role in this disorder (32). GSTM3 is an enzyme that detoxifies electrophilic compounds via the conjugation of GSH (33). The decrease in GSTM3 expression observed in the ASD group in our study suggests that the protective effect of GSH is diminished in ASD. Immune cell dysfunction can lead to the release of proinflammatory mediators such as cytokines, chemokines, and reactive oxygen species (ROS), which are thought to contribute to the pathogenesis of ASD (5, 34, 35). Some inflammatory cytokines and oxidative stress parameters were shown to be positively associated with impaired behavioral patterns in patients with ASD, indicating that antioxidants play an essential role in the modulation of inflammatory potential (36, 37).

Our results showed that OPN, interleukin (IL)-17B, and toll-like receptor 4 interactor with leucine-rich repeats precursor (TRIL) were upregulated in the ASD group. OPN is a proinflammatory cytokine that plays a key role in inflammation, biomineralization, cellular viability, cancer, and diabetes via distinct mechanisms (38). OPN can increase the secretion level of interleukin (IL)-10, interleukin (IL)-12, and nuclear factor (NF)-κB and stimulate IL-17 production, which was shown to be correlated with ASD severity (5). It was also reported that IL-17A/IL-17R activation plays an important role in autism through the upregulation of oxidative and inflammatory mediators (36). We, therefore, speculated that the increased expression of IL-17B in the ASD group resulted from the induction of OPN, which caused oxidative damage and neuroinflammation. In addition, ASD was shown to be associated with increased expression of toll-like receptor (TLR) 4 on T cells (39). TLRs contribute to the pathogenesis of ASD by triggering NADPH oxidase (NOX)-2–derived ROS. In this study, TRIL was upregulated in the ASD group; we speculate that its activation enhances the production of NOX-2–derived ROS.

Tryptophan metabolism is linked to inflammation, the immune response, and oxidative stress in neurovascular diseases (40). We observed that tryptophan metabolism was markedly altered in ASD, consistent with previous studies (41–43) and supporting the role of tryptophan metabolism in neuroinflammation in ASD. Tryptophan can be metabolized in the intestine to form indole and its derivatives that act as aryl hydrocarbon receptor (AhR) ligands and are regulated by proteins in the cytochrome p450 family. The cytochrome P450 metabolic pathway is involved in the etiology of ASD and CYP1B1 is a potential biomarker of the disorder (44). The alteration in the cytochrome P450 metabolic pathway detected in our integrated analysis may lead to changes in AhR signaling, which may in turn cause dysregulation of tryptophan metabolism and neuroimmune dysfunction leading to ASD.

We observed that an integrated pathway related to immune response—namely, leukocyte transendothelial migration—was significantly enriched in the ASD group. Leukocyte transendothelial migration involves the spatiotemporal regulation of adhesion molecules, chemokines, and cytoskeletal regulators and is essential for the innate and adaptive immune responses (45). Four of the DEPs identified in our analysis were related to leukocyte transendothelial migration including vinculin, vascular cell adhesion protein (VCAM)1, cadherin (CDH)5, and guanine nucleotide exchange factor VAV3 isoform 1 (VAV3). Cell adhesion molecules (CAMs) have been implicated in impaired social interaction and communication and stereotyped and repetitive behaviors that characterize ASD (46–48). The protein levels of 5 other adhesion molecules including neuregulin (NRG)3, neural cell adhesion molecule (NCAM)1, CD166 antigen isoform 1 precursor (ALCAM1), cadherin 16 isoform 1 precursor (CDH16), and macrophage migration inhibitory factor (MIF) were significantly altered in the ASD group compared with the TD group. MIF is an immunomodulatory cytokine that promotes inflammation and directional cell migration, inhibits apoptosis, induces the release of pro-inflammatory factors, and may contribute to ASD through the regulation of neuroinflammation (49). Plasma MIF concentration was found to be correlated with ASD severity and MIF may be a susceptibility gene in ASD (50). In addition, serum MIF levels were higher in patients with more severe ASD, and elevated MIF expression was positively correlated with ASD severity (51). In line with these earlier studies, we found that MIF expression was upregulated in children with ASD as compared with those with TD. Taken together, these data indicate that CAMs participate in the induction of neuroinflammation in ASD, although the significance of this effect on ASD development remains to be determined.

A total of 11 immunoglobulins showed significantly altered expression in the ASD group compared with the TD group, consistent with previous findings (52). Two of these immunoglobulins, HLA class I histocompatibility antigen (HLA-A) and zinc-alpha-2-glycoprotein precursor (AZGP1), are known to be involved in antigen processing and presentation, as well as graft-vs. -host disease and, are related to neuroinflammation. HLA is the expression product of the major histocompatibility complex (MHC) and participates in the immune response by presenting peptides derived from the endoplasmic reticulum lumen (53). When the blood–brain barrier is damaged, it is penetrated by immune cells in the peripheral circulation and interacts with glial cells and neurons expressing MHC, resulting in the persistence of the immune response, interfering with the normal development of the nervous system, and leading to ASD.

Prostaglandin E2 (PGE2), an arachidonic acid metabolite of the cyclooxygenase (COX) signaling, is an endogenous lipid molecule that plays a key role during early nervous system development, and PGE2 signaling has been an important factor in the correlation between oxidative stress and ASD (54). Evidence from other studies suggested the link between irregular COX2/PGE2 signaling and autism-related behaviors (55). In addition, PGE2 plays a key role in inflammation in Alzheimer's disease (AD) and shows increased expression in patients with AD compared with the normal subjects (56). In this study, we found that PGE2 was significantly upregulated in the ASD group by metabolomic analysis. Elevated PGE2 levels may also be linked to neuroinflammation in ASD.

## Conclusion

**Figure 8:**
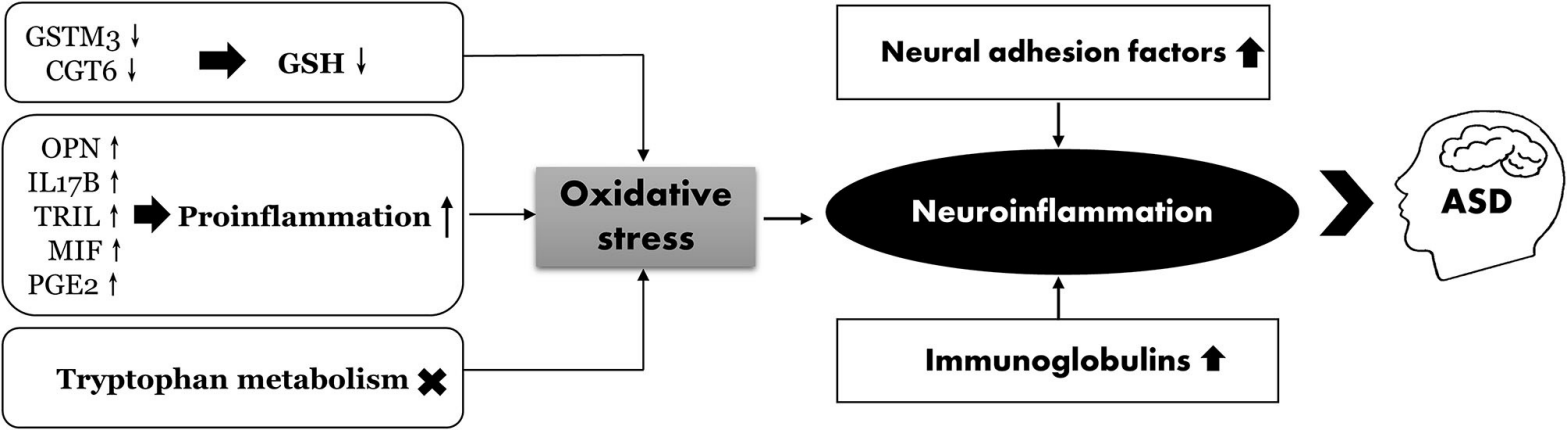
The proposed scheme illustrates neuroinflammation correlated with ASD.

We performed an integrated proteomic and metabolomic analysis to assess the role of neuroimmune responses in the development of ASD. Several metabolic pathways have been identified that may underlie ASD pathogenesis. As shown in Figure 8, oxidative stress may play a crucial role in the development of ASD by suppressing the protective effects of GSH, activating proinflammatory cytokines, and altering tryptophan metabolism. In addition, both the cell adhesion molecules and immunoglobulins can induce neuroinflammation in ASD. We also provide the first evidence of an association between PGE2 levels and inflammation in ASD. These results provide potential biomarkers that can be used for early ASD screening, risk stratification of patients with ASD, and the development of effective and targeted interventions.

## Data Availability Statement

The data presented in the study are deposited in the ProteomeXchange Consortium (http://proteomecentral.proteomexchange.org) via the iProX partner repository (57), accession number PXD031652.

## Ethics Statement

The studies involving human participants were reviewed and approved by the Ethics Committee of Tongji Medical College, Huazhong University of Science and Technology (Hubei, China). Written informed consent to participate in this study was provided by the participants' legal guardian/next of kin.

## Author Contributions

WL and JW designed the experiments and contributed to the critical revision of the article. WL analyzed and drafted the initial manuscript. WL, LL, and YD took part in the collection of urine samples. XX and JW participated in the proteomic analysis. XZ, ZY, and JW performed the metabolomic analysis. All authors contributed significantly to this work and have approved the final manuscript.

## Funding

This work was supported by the National Natural Science Foundation of China (No. 81960290), the Natural Science Foundation of Fujian Province of China (No. 2021J01345), the Scientific and Technological Plan Projects of Xiamen (No. 3502Z20159004), and the Foundation of Xiamen Medical College (Nos. K2016-11 and K2020-01).

## Conflict of Interest

The authors declare that the research was conducted in the absence of any commercial or financial relationships that could be construed as a potential conflict of interest.

## Publisher's Note

All claims expressed in this article are solely those of the authors and do not necessarily represent those of their affiliated organizations, or those of the publisher, the editors and the reviewers. Any product that may be evaluated in this article, or claim that may be made by its manufacturer, is not guaranteed or endorsed by the publisher.

## Supplementary Material

The mass spectrometry proteomics data have been deposited to the ProteomeXchange Consortium (http://proteomecentral.proteomexchange.org) via the iProX partner repository (57) with the dataset identifier PXD031652.

Supplementary Figure 1 Identification of DEPs. The X-axis represents protein difference (log2-transformed fold changes), and the Y-axis the corresponding -log10-transformed *P*-values. Red dots indicate significantly upregulated proteins, green dots indicate significantly downregulated proteins, and gray dots indicate no significant change.

Supplementary Figure 2 Total ion chromatogram of a urine sample. **(A)** positive ion mode; **(B)** negative ion mode.

Supplementary Figure 3 The QC analysis of the proteome. **(A)** within-group coefficient of variation; **(B)** Principal Component Analysis (PCA).

Supplementary Table 1 The significantly enriched terms in the biological process.

Supplementary Table 2 The top 10 different metabolites in positive ion mode.

Supplementary Table 3 The top 10 different metabolites in negative ion mode.

## References

[1] Masi A DeMayo MM Glozier N Guastella AJ. An overview of autism spectrum disorder, heterogeneity and treatment options. Neurosci Bull. (2017) 33:183–93. 10.1007/s12264-017-0100-y 28213805 PMC5360849

[2] Matthew JM Kelly AS Jon B Anita W Mary P Monica D. Prevalence of autism spectrum disorder among children aged 8 years - autism and developmental disabilities monitoring network, 11 sites, United States, 2016. MMWR Surveill Summ. (2020) 69:1–12. 10.15585/mmwr.ss6904a1 32214087 PMC7119644

[3] Zhou H Xu X Yan W Zou X Wu L Luo X. Prevalence of autism spectrum disorder in China: a nationwide multi-center population-based study among children aged 6 to 12 years. Neurosci Bull. (2020) 36:961–71. 10.1007/s12264-020-00530-6 32607739 PMC7475160

[4] Ravaccia D Ghafourian T. Critical role of the maternal immune system in the pathogenesis of autism spectrum disorder. Biomedicines. (2020) 8:557. 10.3390/biomedicines8120557 33271759 PMC7760377

[5] Meltzer A Van de Wate J. The role of the immune system in autism spectrum disorder. Neuropsychopharmacology. (2017) 42:284–98. 10.1038/npp.2016.158 27534269 PMC5143489

[6] Al-ayadhi LY Mostafa GA. Increased serum osteopontin levels in autistic children: relation to the disease severity. Brain Behav Immun. (2011) 25:1393–8. 10.1016/j.bbi.2011.04.006 21521652

[7] Careaga M Rogers S Hansen RL Amaral DG Van de Water J Ashwood P. Immune endophenotypes in children with autism spectrum disorder. Biol Psychiatry. (2017) 81:434–41. 10.1016/j.biopsych.2015.08.036 26493496 PMC4788581

[8] Choi GB Yim YS Wong H Kim S Kim H Kim SV. The maternal interleukin-17a pathway in mice promotes autism-like phenotypes in offspring. Science. (2016) 351:933–9. 10.1126/science.aad0314 26822608 PMC4782964

[9] Grzadzinski R Lord C Sanders SJ Werling D Bal VH. Children with autism spectrum disorder who improve with fever: insights from the simons simplex collection. Autism Res. (2018) 11:175–84. 10.1002/aur.1856 28861935

[10] Shen L Feng C Zhang K Chen Y Gao Y Ke J. Proteomics study of peripheral blood mononuclear cells (PBMCs) in autistic children. Front Cell Neurosci. (2019) 13:105. 10.3389/fncel.2019.00105 30941018 PMC6433831

[11] Abraham JR Szoko N Barnard J Rubin RA Schlatzer D Lundeberg K. Proteomic investigations of autism brain identify known and novel pathogenetic processes. Sci Rep. (2019) 9:13118. 10.1038/s41598-019-49533-y 31511657 PMC6739414

[12] Ming X Stein TP Barnes V Rhodes N Guo L. Metabolic perturbance in autism spectrum disorders: a metabolomics study. J Proteome Res. (2012) 11:5856–62. 10.1021/pr300910n 23106572

[13] Agus A Planchais J Sokol H. Gut microbiota regulation of tryptophan metabolism in health and disease. Cell Host Microbe. (2018) 23:716–24. 10.1016/j.chom.2018.05.003 29902437

[14] Graham SF Turkoglu O Yilmaz A Ustun I Ugur Z Bjorndhal T. Targeted metabolomics highlights perturbed metabolism in the brain of autism spectrum disorder sufferers. Metabolomics. (2020) 16:59. 10.1007/s11306-020-01685-z 32333121

[15] Wu ZL Chen SY Hu SQ Jia XB Wang J Lai SJ. Metabolomic and proteomic profiles associated with ketosis in dairy cows. Front Genet. (2020) 11:551587. 10.3389/fgene.2020.551587 33391334 PMC7772412

[16] Wang XQ Li FJ Liu L Ji CL Wu HF. Transcriptomic, proteomic and metabolomic profiling unravel the mechanisms of hepatotoxicity pathway induced by triphenyl phosphate (TPP). Ecotoxicol Environ Saf. (2020) 205:111126. 10.1016/j.ecoenv.2020.111126 32823070

[17] Wang JP Liu Y Zhao GX Gao JY Liu JL Wu XR. Integrated proteomic and metabolomic analysis to study the effects of spaceflight on Candida albicans. BMC Genomics. (2020) 21:57. 10.1186/s12864-020-6476-5 31952470 PMC6969454

[18] Walker ME Song RJ Xu X Gerszten RE Ngo D Clish CB. Proteomic and metabolomic correlates of healthy dietary patterns: the framingham heart study. Nutrients. (2020) 12:1476. 10.3390/nu12051476 32438708 PMC7284467

[19] American Psychiatric Association. Diagnostic and Statistical Manual of Mental Disorders (DSM-5. 5th ed. Arlington: American Psychiatric Publishing (2013).

[20] Ma J Liu M Wang Y Xin C Zhang H Chen S. Quantitative proteomics analysis of young and elderly skin with DIA mass spectrometry reveals new skin aging-related proteins. Aging. (2020) 12:13529–54. 10.18632/aging.103461 32602849 PMC7377841

[21] Muntel J Xuan Y Berger ST Reiter L Bachur R Kentsis A. Advancing urinary protein biomarker discovery by data-independent acquisition on a quadrupole-orbitrap mass spectrometer. J Proteome Res. (2015) 14:4752–62. 10.1021/acs.jproteome.5b00826 26423119 PMC4993212

[22] Bruderer R Bernhardt OM Gandhi T Miladinovic SM Cheng LY Messner S. Extending the limits of quantitative proteome profiling with data-independent acquisition and application to acetaminophen-treated three-dimensional liver microtissues. Mol Cell Proteomics. (2015) 14:1400–10. 10.1074/mcp.M114.044305 25724911 PMC4424408

[23] Li YT Wang DM Zeng CW Liu YC Huang GY Mei ZL. Salivary metabolomics profile of patients with recurrent aphthous ulcer as revealed by liquid chromatography-tandem mass spectrometry. J Int Med Res. (2018) 46:1052–62. 10.1177/0300060517745388 29332424 PMC5972264

[24] Wu JL Zhou CX Wu PJ Xu J Guo YQ Fei X. Brain metabolomic profiling of eastern honey bee (Apis cerana) infested with the mite Varroa destructor. PLoS ONE. (2017) 12:e0175573. 10.1371/journal.pone.0175573 28403242 PMC5389839

[25] Liu RX Hong J Xu XQ Feng Q Zhang DY Gu YY. Gut microbiome and serum metabolome alterations in obesity and after weight-loss intervention. Nat Med. (2017) 23:859–68. 10.1038/nm.4358 28628112

[26] Di Guida R Engel J Allwood JW Weber RJ Jones MR Sommer U. Non-targeted UHPLC-MS metabolomic data processing methods: a comparative investigation of normalisation, missing value imputation, transformation and scaling. Metabolomics. (2016) 12:93. 10.1007/s11306-016-1030-9 27123000 PMC4831991

[27] Lee KR Lin X Park DC Eslava S. Megavariate data analysis of mass spectrometric proteomics data using latent variable projection method. Proteomics. (2003) 3:1680–6. 10.1002/pmic.200300515 12973725

[28] Xia JG Wishart DS. Using metaboanalyst 3.0 for comprehensive metabolomics data analysis. Curr Protoc Bioinformatics. (2016) 55:14.10.1–14.10.91. 10.1002/cpbi.11 27603023

[29] Chong JM Wishart DS Xia JG. Using metaboanalyst 4.0 for comprehensive and integrative metabolomics data analysis. Curr Protoc Bioinformatics. (2019) 68:e86. 10.1002/cpbi.86 31756036

[30] Bjorklund G Dosa MD Maes M Dadar M Frye RE Peana M. The impact of glutathione metabolism in autism spectrum disorder. Pharmacol Res. (2021) 166:105437. 10.1016/j.phrs.2021.105437 33493659

[31] Chen L Shi XJ Liu H Mao X Gui LN Wang H. Oxidative stress marker aberrations in children with autism spectrum disorder: a systematic review and meta-analysis of 87 studies (N = 9109). Transl Psychiatry. (2021) 11:15. 10.1038/s41398-020-01135-3 33414386 PMC7791110

[32] Dong D Zielke HR Yeh D Yang P. Cellular stress and apoptosis contribute to the pathogenesis of autism spectrum disorder. Autism Res. (2018) 11:1076–90. 10.1002/aur.1966 29761862 PMC6107407

[33] Lin JH Tu SH Chen LC Huang CC Chang HL Cheng TC. Oestrogen receptor-regulated glutathione S-transferase mu 3 expression attenuates hydrogen peroxide-induced cytotoxicity, which confers tamoxifen resistance on breast cancer cells. Breast Cancer Res Treat. (2018) 172:45–59. 10.1007/s10549-018-4897-5 30054830

[34] Bilbo SD Schwarz JM. The immune system and developmental programming of brain and behavior. Front Neuroendocrinol. (2012) 33:267–86. 10.1016/j.yfrne.2012.08.006 22982535 PMC3484177

[35] Liu A Zhou W Qu L He F Wang H Wang Y. Altered urinary amino acids in children with autism spectrum disorders. Front Cell Neurosci. (2019) 13:7. 10.3389/fncel.2019.00007 30733669 PMC6354128

[36] Nadeem A Ahmad SF Attia SM Al-Ayadhi LY Bakheet SA Al-Harbi NO. Oxidative and inflammatory mediators are upregulated in neutrophils of autistic children: role of IL-17A receptor signaling. Prog Neuropsychopharmacol Biol Psychiatry. (2019) 90:204–11. 10.1016/j.pnpbp.2018.12.002 30529000

[37] Nadeem A Ahmad SF Attia SM Al-Ayadhi LY Al-Harbi NO Bakheet SA. Dysregulated enzymatic antioxidant network in peripheral neutrophils and monocytes in children with autism. Prog Neuropsychopharmacol Biol Psychiatry. (2019) 88:352–9. 10.1016/j.pnpbp.2018.08.020 30145184

[38] Icer MA Gezmen-Karadag M. The multiple functions and mechanisms of osteopontin. Clin Biochem. (2018) 59:17–24. 10.1016/j.clinbiochem.2018.07.003 30003880

[39] Nadeem A Ahmad SF Bakheet SA Al-Harbi NO Al-Ayadhi LY Attia SM. Toll-like receptor 4 signaling is associated with upregulated NADPH oxidase expression in peripheral T cells of children with autism. Brain Behav Immun. (2017) 61:146–54. 10.1016/j.bbi.2016.12.024 28034626

[40] Hajsl M Hlavackova A Broulikova K Sramek M Maly M Dyr JE. Tryptophan metabolism, inflammation, and oxidative stress in patients with neurovascular disease. Metabolites. (2020) 10:208. 10.3390/metabo10050208 32438592 PMC7281607

[41] Gevi F Zolla L Gabriele S Persico AM. Urinary metabolomics of young Italian autistic children supports abnormal tryptophan and purine metabolism. Mol Autism. (2016) 7:47. 10.1186/s13229-016-0109-5 27904735 PMC5121959

[42] Mavel S Nadal-Desbarats L Blasco H Bonnet-Brilhault F Barthelemy C Montigny F. 1H-13C NMR-based urine metabolic profiling in autism spectrum disorders. Talanta. (2013) 114:95–102. 10.1016/j.talanta.2013.03.064 23953447

[43] Kaluzna-Czaplinska J. Noninvasive urinary organic acids test to assess biochemical and nutritional individuality in autistic children. Clin Biochem. (2011) 44:686–91. 10.1016/j.clinbiochem.2011.01.015 21300048

[44] El-Ansary A Cannell JJ Bjorklund G Bhat RS Al Dbass AM Alfawaz HA. In the search for reliable biomarkers for the early diagnosis of autism spectrum disorder: the role of vitamin D. Metab Brain Dis. (2018) 33:917–31. 10.1007/s11011-018-0199-1 29497932

[45] Worthylake RA Burridge K. Leukocyte transendothelial migration: orchestrating the underlying molecular machinery. Curr Opin Cell Biol. (2001) 13:569–77. 10.1016/S0955-0674(00)00253-2 11544025

[46] Feng J Schroer R Yan J Song W Yang C Bockholt A. High frequency of neurexin 1beta signal peptide structural variants in patients with autism. Neurosci Lett. (2006) 409:10–13. 10.1016/j.neulet.2006.08.017 17034946

[47] Sanders SJ Ercan-Sencicek AG Hus V Luo R Murtha MT Moreno-De-Luca D. et al. Multiple recurrent de novo CNVs, including duplications of the 7q1123 Williams syndrome region, are strongly associated with autism. Neuron. (2011) 70:863–85. 10.1016/j.neuron.2011.05.002 21658581 PMC3939065

[48] van Daalen E Kemner C Verbeek NE van der Zwaag B Dijkhuizen T Rump P. Social responsiveness scale-aided analysis of the clinical impact of copy number variations in autism. Neurogenetics. (2011) 12:315–23. 10.1007/s10048-011-0297-2 21837366 PMC3215885

[49] Leyton-Jaimes MF Kahn J Israelson A. Macrophage migration inhibitory factor: a multifaceted cytokine implicated in multiple neurological diseases. Exp Neurol. (2018) 301:83–91. 10.1016/j.expneurol.2017.06.021 28679106

[50] Grigorenko EL Han SS Yrigollen CM Leng L Mizue Y Anderson GM. Macrophage migration inhibitory factor and autism spectrum disorders. Pediatrics. (2008) 122:e438–45. 10.1542/peds.2007-3604 18676531 PMC3816765

[51] Ning J Xu L Shen CQ Zhang YY Zhao Q. Increased serum levels of macrophage migration inhibitory factor in autism spectrum disorders. Neurotoxicology. (2019) 71:1–5. 10.1016/j.neuro.2018.11.015 30503813

[52] Ngounou Wetie AG Wormwood KL Russell S Ryan JP Darie CC Woods AG. Pilot proteomic analysis of salivary biomarkers in autism spectrum disorder. Autism Res. (2015) 8:338–50. 10.1002/aur.1450 25626423

[53] Torres MA Moraes ME. Nomenclature for factors of the HLA system. Einstein. (2011) 9:249–51. 10.1590/s1679-45082011md1914 26760826

[54] De Felice A Greco A Calamandrei G Minghetti L. Prenatal exposure to the organophosphate insecticide chlorpyrifos enhances brain oxidative stress and prostaglandin E2 synthesis in a mouse model of idiopathic autism. J Neuroinflammation. (2016) 13:149. 10.1186/s12974-016-0617-4 27301868 PMC4908699

[55] Wong CT Bestard-Lorigados I Crawford DA. Autism-related behaviors in the cyclooxygenase-2-deficient mouse model. Genes Brain Behav. (2019) 18:e12506. 10.1111/gbb.12506 30027581

[56] Montine TJ Sidell KR Crews BC Markesbery WR Marnett LJ Roberts LJ. Elevated CSF prostaglandin E2 levels in patients with probable AD. Neurology. (1999) 53:1495–8. 10.1212/WNL.53.7.1495 10534257

[57] Ma J Chen T Wu S Yang C Bai M Shu K. iProX: an integrated proteome resource. Nucleic Acids Res. (2019) 47:D1211–7. 10.1093/nar/gky869 30252093 PMC6323926

