# Supplementary material for: Integration of Urine Proteomic and Metabolomic Profiling Reveals Novel Insights Into Neuroinflammation in Autism Spectrum Disorder
Source: Front Psychiatry. 2022 May 9;13:780747. doi: 10.3389/fpsyt.2022.780747 (PMC9124902; doi:10.3389/fpsyt.2022.780747)
Supplement: Supplementary Figure 1 — Identification of DEPs. The X-axis represents protein difference (log2-transformed fold changes), and the Y-axis the corresponding -log10-transformed P-values. Red dots indicate significantly upregulated proteins, green dots indicate significantly downregulated proteins, and gray dots indicate no significant change. [file Data_Sheet_1.zip › Table S1.docx]

**Table S1 The significant enriched terms in biological process**

| **GO ID** | **GO name** | **Count** | ***p* value** |
| --- | --- | --- | --- |
| GO:0032501 | Multicellular organismal process | 42 | 0.018 |
| GO:0032502 | Developmental process | 37 | 0.019 |
| GO:0048856 | Anatomical structure development | 34 | 0.049 |
| GO:0007275 | Multicellular organism development | 33 | 0.029 |
| GO:0002376 | Immune system process | 28 | 0.031 |
| GO:0006955 | Immune response | 25 | 0.005 |
| GO:0051239 | Regulation of multicellular organismal process | 25 | 0.034 |
| GO:0001775 | Cell activation | 20 | 0.015 |
| GO:0002252 | Immune effector process | 19 | 0.020 |
| GO:0032940 | Secretion by cell | 19 | 0.037 |
| GO:0045321 | Leukocyte activation | 18 | 0.017 |
| GO:0009605 | Response to external stimulus | 18 | 0.021 |
| GO:0002443 | Leukocyte mediated immunity | 17 | 0.023 |
| GO:0045055 | Regulated exocytosis | 17 | 0.038 |
| GO:0006887 | Exocytosis | 17 | 0.046 |
| GO:0043299 | Leukocyte degranulation | 16 | 0.014 |
| GO:0002275 | Myeloid cell activation involved in immune response | 16 | 0.016 |
| GO:0002444 | Myeloid leukocyte mediated immunity | 16 | 0.016 |
| GO:0002274 | Myeloid leukocyte activation | 16 | 0.019 |
| GO:0002366 | Leukocyte activation involved in immune response | 16 | 0.021 |
| GO:0002263 | Cell activation involved in immune response | 16 | 0.023 |
| GO:0051240 | Positive regulation of multicellular organismal process | 16 | 0.045 |
| GO:0002283 | Neutrophil activation involved in immune response | 15 | 0.026 |
| GO:0042119 | Neutrophil activation | 15 | 0.026 |
| GO:0043312 | Neutrophil degranulation | 15 | 0.026 |
| GO:0036230 | Granulocyte activation | 15 | 0.027 |
| GO:0002446 | Neutrophil mediated immunity | 15 | 0.030 |
| GO:0051241 | Negative regulation of multicellular organismal process | 14 | 0.018 |
| GO:0006952 | Defense response | 14 | 0.030 |
| GO:0035556 | Intracellular signal transduction | 13 | 0.006 |
| GO:0006954 | Inflammatory response | 10 | 0.004 |
| GO:0005975 | Carbohydrate metabolic process | 9 | 0.023 |
| GO:0009617 | Response to bacterium | 8 | 0.035 |
| GO:0090066 | Regulation of anatomical structure size | 8 | 0.035 |
| GO:0006935 | Chemotaxis | 8 | 0.048 |
| GO:0042330 | Taxis | 8 | 0.048 |
| GO:0007568 | Aging | 7 | 0.003 |
| GO:0044706 | Multi-multicellular organism process | 6 | 0.005 |
| GO:0019318 | Hexose metabolic process | 6 | 0.011 |
| GO:0002237 | Response to molecule of bacterial origin | 6 | 0.015 |
| GO:1901136 | Carbohydrate derivative catabolic process | 6 | 0.022 |
| GO:0005996 | Monosaccharide metabolic process | 6 | 0.024 |
| GO:0051222 | Positive regulation of protein transport | 6 | 0.049 |
| GO:0006027 | Glycosaminoglycan catabolic process | 5 | 0.010 |
| GO:0006026 | Aminoglycan catabolic process | 5 | 0.012 |
| GO:0007565 | Female pregnancy | 5 | 0.016 |
| GO:0032844 | Regulation of homeostatic process | 5 | 0.030 |
| GO:0032496 | Response to lipopolysaccharide | 5 | 0.039 |
| GO:0030203 | Glycosaminoglycan metabolic process | 5 | 0.046 |
| GO:0006022 | Aminoglycan metabolic process | 5 | 0.049 |
| GO:0030279 | Negative regulation of ossification | 4 | 0.000 |
| GO:0002700 | Regulation of production of molecular mediator of immune response | 4 | 0.005 |
| GO:0008016 | Regulation of heart contraction | 4 | 0.013 |
| GO:0019932 | Second-messenger-mediated signaling | 4 | 0.018 |
| GO:0003012 | Muscle system process | 4 | 0.020 |
| GO:0030278 | Regulation of ossification | 4 | 0.020 |
| GO:1903522 | Regulation of blood circulation | 4 | 0.033 |
| GO:0002526 | Acute inflammatory response | 4 | 0.037 |
| GO:0007586 | Digestion | 4 | 0.037 |
| GO:0050714 | Positive regulation of protein secretion | 4 | 0.045 |
| GO:0030816 | Positive regulation of cAMP metabolic process | 3 | 0.001 |
| GO:0030801 | Positive regulation of cyclic nucleotide metabolic process | 3 | 0.002 |
| GO:0045823 | Positive regulation of heart contraction | 3 | 0.003 |
| GO:0060333 | Interferon-gamma-mediated signaling pathway | 3 | 0.003 |
| GO:0019933 | cAMP-mediated signaling | 3 | 0.007 |
| GO:0019935 | Cyclic-nucleotide-mediated signaling | 3 | 0.007 |
| GO:0002702 | Positive regulation of production of molecular mediator of immune response | 3 | 0.009 |
| GO:0030814 | Regulation of cAMP metabolic process | 3 | 0.009 |
| GO:0050871 | Positive regulation of B cell activation | 3 | 0.009 |
| GO:0002718 | Regulation of cytokine production involved in immune response | 3 | 0.012 |
| GO:0050906 | Detection of stimulus involved in sensory perception | 3 | 0.012 |
| GO:1903524 | Positive regulation of blood circulation | 3 | 0.012 |
| GO:0002027 | Regulation of heart rate | 3 | 0.015 |
| GO:0007566 | Embryo implantation | 3 | 0.015 |
| GO:0030799 | Regulation of cyclic nucleotide metabolic process | 3 | 0.015 |
| GO:0032760 | Positive regulation of tumor necrosis factor production | 3 | 0.015 |
| GO:0045981 | Positive regulation of nucleotide metabolic process | 3 | 0.015 |
| GO:0071346 | Cellular response to interferon-gamma | 3 | 0.015 |
| GO:1900544 | Positive regulation of purine nucleotide metabolic process | 3 | 0.015 |
| GO:0034341 | Response to interferon-gamma | 3 | 0.018 |
| GO:1903557 | Positive regulation of tumor necrosis factor superfamily cytokine production | 3 | 0.018 |
| GO:0050864 | Regulation of B cell activation | 3 | 0.022 |
| GO:0006953 | Acute-phase response | 3 | 0.036 |
| GO:0033500 | Carbohydrate homeostasis | 3 | 0.041 |
| GO:0042593 | Glucose homeostasis | 3 | 0.041 |
| GO:0050715 | Positive regulation of cytokine secretion | 3 | 0.041 |
| GO:0006004 | Fucose metabolic process | 2 | 0.002 |
| GO:0008209 | Androgen metabolic process | 2 | 0.005 |
| GO:0042181 | Ketone biosynthetic process | 2 | 0.005 |
| GO:0044245 | Polysaccharide digestion | 2 | 0.005 |
| GO:0045879 | Negative regulation of smoothened signaling pathway | 2 | 0.005 |
| GO:0050716 | Positive regulation of interleukin-1 secretion | 2 | 0.005 |
| GO:0050718 | Positive regulation of interleukin-1 beta secretion | 2 | 0.005 |
| GO:1904467 | Regulation of tumor necrosis factor secretion | 2 | 0.005 |
| GO:1904469 | Positive regulation of tumor necrosis factor secretion | 2 | 0.005 |
| GO:0006907 | Pinocytosis | 2 | 0.010 |
| GO:0010460 | Positive regulation of heart rate | 2 | 0.010 |
| GO:0032878 | Regulation of establishment or maintenance of cell polarity | 2 | 0.010 |
| GO:0050706 | Regulation of interleukin-1 beta secretion | 2 | 0.010 |
| GO:2000114 | Regulation of establishment of cell polarity | 2 | 0.010 |
| GO:0016139 | Glycoside catabolic process | 2 | 0.016 |
| GO:0030819 | Positive regulation of cAMP biosynthetic process | 2 | 0.016 |
| GO:0032731 | Positive regulation of interleukin-1 beta production | 2 | 0.016 |
| GO:0070229 | Negative regulation of lymphocyte apoptotic process | 2 | 0.016 |
| GO:0002026 | Regulation of the force of heart contraction | 2 | 0.023 |
| GO:0002720 | Positive regulation of cytokine production involved in immune response | 2 | 0.023 |
| GO:0007569 | Cell aging | 2 | 0.023 |
| GO:0021695 | Cerebellar cortex development | 2 | 0.023 |
| GO:0030804 | Positive regulation of cyclic nucleotide biosynthetic process | 2 | 0.023 |
| GO:0032732 | Positive regulation of interleukin-1 production | 2 | 0.023 |
| GO:0045124 | Regulation of bone resorption | 2 | 0.023 |
| GO:0048286 | Lung alveolus development | 2 | 0.023 |
| GO:0050704 | Regulation of interleukin-1 secretion | 2 | 0.023 |
| GO:0007189 | Adenylate cyclase-activating G-protein coupled receptor signaling pathway | 2 | 0.031 |
| GO:0030890 | Positive regulation of B cell proliferation | 2 | 0.031 |
| GO:0032651 | Regulation of interleukin-1 beta production | 2 | 0.031 |
| GO:0033574 | Response to testosterone | 2 | 0.031 |
| GO:0035725 | Sodium ion transmembrane transport | 2 | 0.031 |
| GO:0035924 | Cellular response to vascular endothelial growth factor stimulus | 2 | 0.031 |
| GO:0046850 | Regulation of bone remodeling | 2 | 0.031 |
| GO:0048675 | Axon extension | 2 | 0.031 |
| GO:1900076 | Regulation of cellular response to insulin stimulus | 2 | 0.031 |
| GO:0008589 | Regulation of smoothened signaling pathway | 2 | 0.040 |
| GO:0016137 | Glycoside metabolic process | 2 | 0.040 |
| GO:0019233 | Sensory perception of pain | 2 | 0.040 |
| GO:0030032 | Lamellipodium assembly | 2 | 0.040 |
| GO:0030810 | Positive regulation of nucleotide biosynthetic process | 2 | 0.040 |
| GO:0032370 | Positive regulation of lipid transport | 2 | 0.040 |
| GO:0032715 | Negative regulation of interleukin-6 production | 2 | 0.040 |
| GO:0070228 | Regulation of lymphocyte apoptotic process | 2 | 0.040 |
| GO:1900373 | Positive regulation of purine nucleotide biosynthetic process | 2 | 0.040 |

*GO: Gene Ontology*
