# Supplementary material for: Integration of Urine Proteomic and Metabolomic Profiling Reveals Novel Insights Into Neuroinflammation in Autism Spectrum Disorder
Source: Front Psychiatry. 2022 May 9;13:780747. doi: 10.3389/fpsyt.2022.780747 (PMC9124902; doi:10.3389/fpsyt.2022.780747)
Supplement: Supplementary Figure 1 — Identification of DEPs. The X-axis represents protein difference (log2-transformed fold changes), and the Y-axis the corresponding -log10-transformed P-values. Red dots indicate significantly upregulated proteins, green dots indicate significantly downregulated proteins, and gray dots indicate no significant change. [file Data_Sheet_1.zip › Table S2.docx]

**Table S2. The top 10 different metabolites in positive ion mode**

| **Compound ID** | **Metabolites** | **Class** | **m.z** | **Ratio** | ***q-*value** | **VIP** |
| --- | --- | --- | --- | --- | --- | --- |
| 3.86_214.1551m/z | Guanadrel Sulfate | Organooxygen compounds | 214.16 | 18.13↑ | 0.018 | 2.56 |
| 7.20_370.2952m/z | MG (18:3(6Z,9Z,12Z)/0:0/0:0) | Fatty Acyls | 370.30 | 6.03↑ | 0.016 | 2.90 |
| 4.47_638.3603m/z | Allodesmosine | Carboxylic acids and derivatives | 638.36 | 3.49↑ | 0.000 | 4.02 |
| 5.47_370.2588m/z | Sterebin B | Prenol lipids | 370.26 | 3.14↑ | 0.008 | 2.10 |
| 3.34_366.2022m/z | Enalaprilat | Carboxylic acids and derivatives | 366.20 | 3.05↑ | 0.031 | 2.32 |
| 6.40_532.3119m/z | Capsianoside V | Prenol lipids | 532.31 | 2.97↑ | 0.015 | 2.67 |
| 7.38_372.3111m/z | MG (0:0/18:2(9Z,12Z)/0:0) | Fatty Acyls | 372.31 | 2.93↑ | 0.007 | 2.52 |
| 4.82_287.0548m/z | Cernuine | Aurone flavonoids | 287.05 | 2.90↑ | 0.046 | 1.77 |
| 2.19_268.1043m/z | Adenosine | Purine nucleosides | 268.10 | 2.65↑ | 0.000 | 3.12 |
| 6.30_181.0861m/z | 2-hydroxy-3-(4-methoxyphenyl) propanal | Phenol ethers | 181.09 | 2.54↑ | 0.005 | 2.57 |
| 2.43_180.0881m/z | (-)-1-Methylpropyl 1-propenyl disulfide | Organic disulfides | 180.09 | 0.06↓ | 0.000 | 3.73 |
| 7.95_325.2740m/z | (13R,14R)-7-Labdene-13,14,15-triol | Prenol lipids | 325.27 | 0.09↓ | 0.010 | 2.98 |
| 7.48_390.2771n | 12alpha-hydroxy-3-oxo-5beta-cholan-24-oic Acid | Steroids and steroid derivatives | 391.28 | 0.12↓ | 0.043 | 2.43 |
| 6.69_262.1208n | Enokipodin D | Prenol lipids | 245.12 | 0.12↓ | 0.029 | 2.52 |
| 5.02_234.9934m/z | p-Chlorobenzene sulfonyl urea | Benzene and substituted derivatives | 234.99 | 0.17↓ | 0.003 | 2.82 |
| 8.08_466.2927m/z | LysoPC(14:1(9Z)) | Glycerophospholipids | 466.29 | 0.19↓ | 0.003 | 2.43 |
| 4.41_206.0562m/z | Nitroxoline | Quinolines and derivatives | 206.06 | 0.20↓ | 0.016 | 2.24 |
| 7.19_276.1365n | Acetylpterosin C | Indanes | 277.14 | 0.23↓ | 0.000 | 2.92 |
| 3.58_153.0546m/z | Herierin IV | Pyrans | 153.05 | 0.26↓ | 0.031 | 2.19 |
| 7.19_322.2014m/z | 2-Polyprenyl-3-methyl-5-hydroxy-6-methoxy-1,4-benzoquinone | Prenol lipids | 322.20 | 0.27↓ | 0.006 | 2.98 |
