# Supplementary material for: Integration of Urine Proteomic and Metabolomic Profiling Reveals Novel Insights Into Neuroinflammation in Autism Spectrum Disorder
Source: Front Psychiatry. 2022 May 9;13:780747. doi: 10.3389/fpsyt.2022.780747 (PMC9124902; doi:10.3389/fpsyt.2022.780747)
Supplement: Supplementary Figure 1 — Identification of DEPs. The X-axis represents protein difference (log2-transformed fold changes), and the Y-axis the corresponding -log10-transformed P-values. Red dots indicate significantly upregulated proteins, green dots indicate significantly downregulated proteins, and gray dots indicate no significant change. [file Data_Sheet_1.zip › Table S3.docx]

**Table S3. The top 10 different metabolites in negative ion mode**

| **Compound ID** | **Metabolites** | **Class** | **m.z** | **Ratio** | ***q-*value** | **VIP** |
| --- | --- | --- | --- | --- | --- | --- |
| 4.08_383.1917m/z | 1,9-Nonanedithiol | Thiols | 383.19 | 4.54↑ | 0.014 | 2.52 |
| 3.31_345.1187m/z | (1xi,2xi)-1-(4-Hydroxyphenyl)-1,2,3-propanetriol 3-O-beta-D-Glucopyranoside | Fatty Acyls | 345.12 | 4.52↑ | 0.039 | 2.22 |
| 2.31_234.9913m/z | 3,5-dihydroxy-4-(sulfooxy) cyclohex-1-ene-1-carboxylic acid | Organic sulfuric acids  and derivatives | 234.99 | 3.46↑ | 0.013 | 2.12 |
| 4.11_180.0663m/z | 2-Acetyl-1,5,6,7-tetrahydro-6-hydroxy-7-(hydroxymethyl)-4H-azepine-4-one | Azepines | 180.07 | 3.30↑ | 0.008 | 2.61 |
| 1.44_387.0731m/z | 3,4,5-trihydroxy-6-{2-[(4-hydroxyphenoxy)carbonyl]phenoxy}oxane-2-carboxylic acid | Tannins | 387.07 | 3.30↑ | 0.011 | 2.61 |
| 0.52_181.0712m/z | 1-Deoxy-D-ribitol | Organooxygen compounds | 181.07 | 3.22↑ | 0.003 | 2.68 |
| 2.85_294.0650m/z | (S)-5'-Deoxy-5'-(methylsulfinyl)adenosine | 5'-deoxyribonucleosides | 294.06 | 2.90↑ | 0.049 | 1.64 |
| 5.18_225.0765m/z | 4-Hydroxy-2,6,6-trimethyl-3-oxo-1,4-cyclohexadiene-1-carboxaldehyde | Organooxygen compounds | 225.08 | 2.71↑ | 0.001 | 3.09 |
| 0.52_219.0456m/z | 7-hydroxy-2-phenyl-4H-chromen-4-one | Flavonoids | 219.05 | 2.67↑ | 0.011 | 2.24 |
| 2.70_218.1044m/z | Cis-zeatin | Fatty Acyls | 218.10 | 2.65↑ | 0.044 | 1.96 |
| 4.21_131.0711m/z | 2-Hydroxycaproic acid | Fatty Acyls | 131.07 | 0.07↓ | 0.001 | 3.63 |
| 2.43_178.0735m/z | 7-Aminomethyl-7-carbaguanine | Pyrrolopyrimidines | 178.07 | 0.07↓ | 0.001 | 3.39 |
| 2.18_149.0464m/z | 7-Methylhypoxanthine | Imidazopyrimidines | 149.05 | 0.11↓ | 0.000 | 3.80 |
| 7.89_391.2846m/z | 7b,12a-Dihydroxycholanoic acid | Glycerophospholipids | 391.28 | 0.20↓ | 0.011 | 3.16 |
| 3.58_106.0416n | Benzaldehyde | Benzene and substituted derivatives | 211.06 | 0.21↓ | 0.038 | 2.34 |
| 0.97_305.0177m/z | 3'-UMP | Ribonucleoside 3'-phosphates | 305.02 | 0.22↓ | 0.004 | 2.97 |
| 0.88_89.0240m/z | 2,2-Dihydroperoxypropane | Organic hydroperoxides | 89.02 | 0.26↓ | 0.000 | 2.96 |
| 1.32_117.0189m/z | Threonic acid | Organooxygen compounds | 117.02 | 0.30↓ | 0.009 | 2.21 |
| 5.43_271.1182m/z | Allixin | Pyrans | 271.12 | 0.37↓ | 0.008 | 2.77 |
| 2.38_265.0924m/z | {[(2Z)-2-(phenylmethylidene) heptyl]oxy}sulfonic acid | Benzene and substituted derivatives | 265.09 | 0.42↓ | 0.001 | 2.69 |
