# Supplementary figures and images for: Integration of Urine Proteomic and Metabolomic Profiling Reveals Novel Insights Into Neuroinflammation in Autism Spectrum Disorder
Source: Front Psychiatry. 2022 May 9;13:780747. doi: 10.3389/fpsyt.2022.780747 (PMC9124902; doi:10.3389/fpsyt.2022.780747)

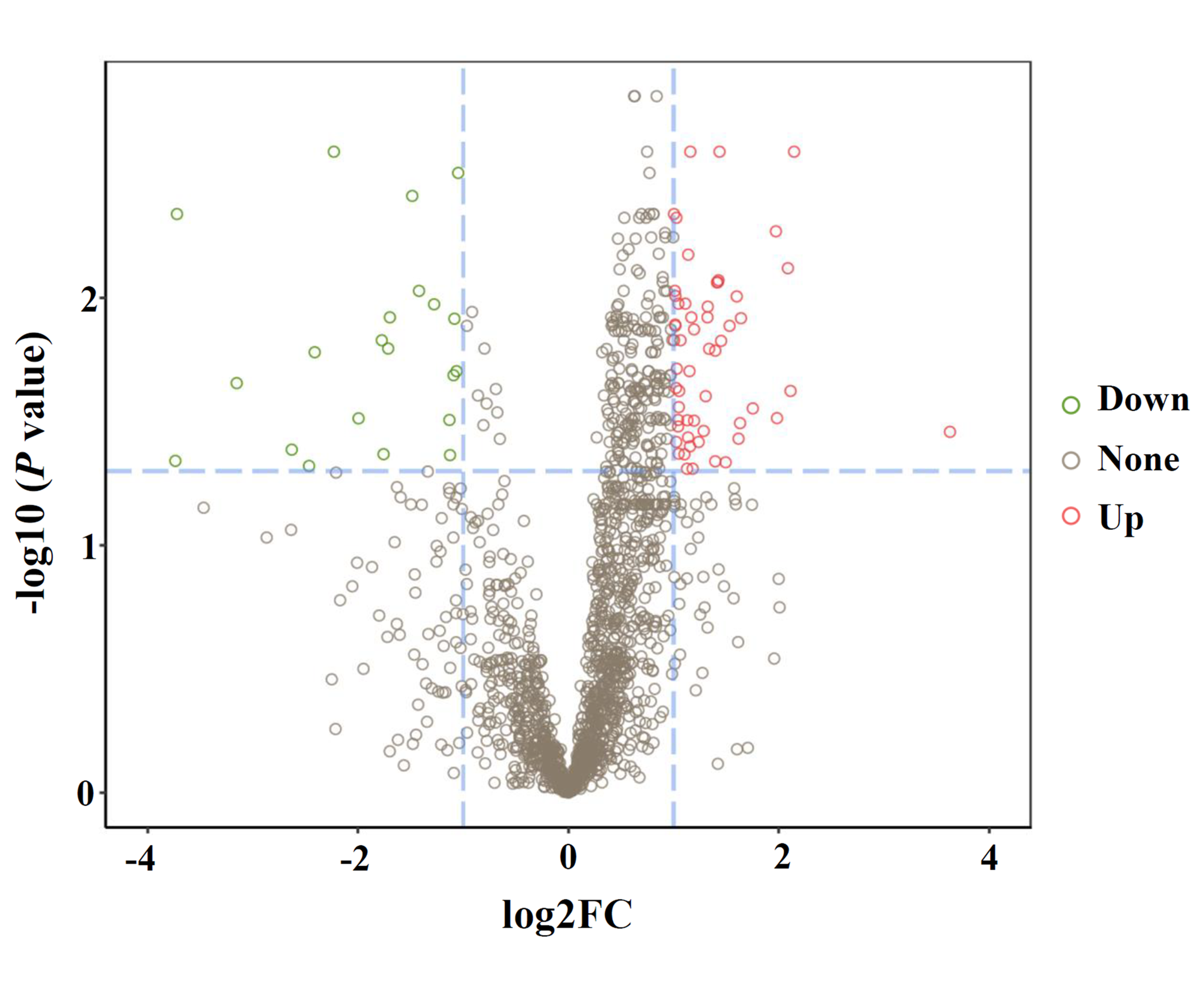

Supplement: Supplementary Figure 1 — Identification of DEPs. The X-axis represents protein difference (log2-transformed fold changes), and the Y-axis the corresponding -log10-transformed P-values. Red dots indicate significantly upregulated proteins, green dots indicate significantly downregulated proteins, and gray dots indicate no significant change. [file Data_Sheet_1.zip › Figure S1.tif]

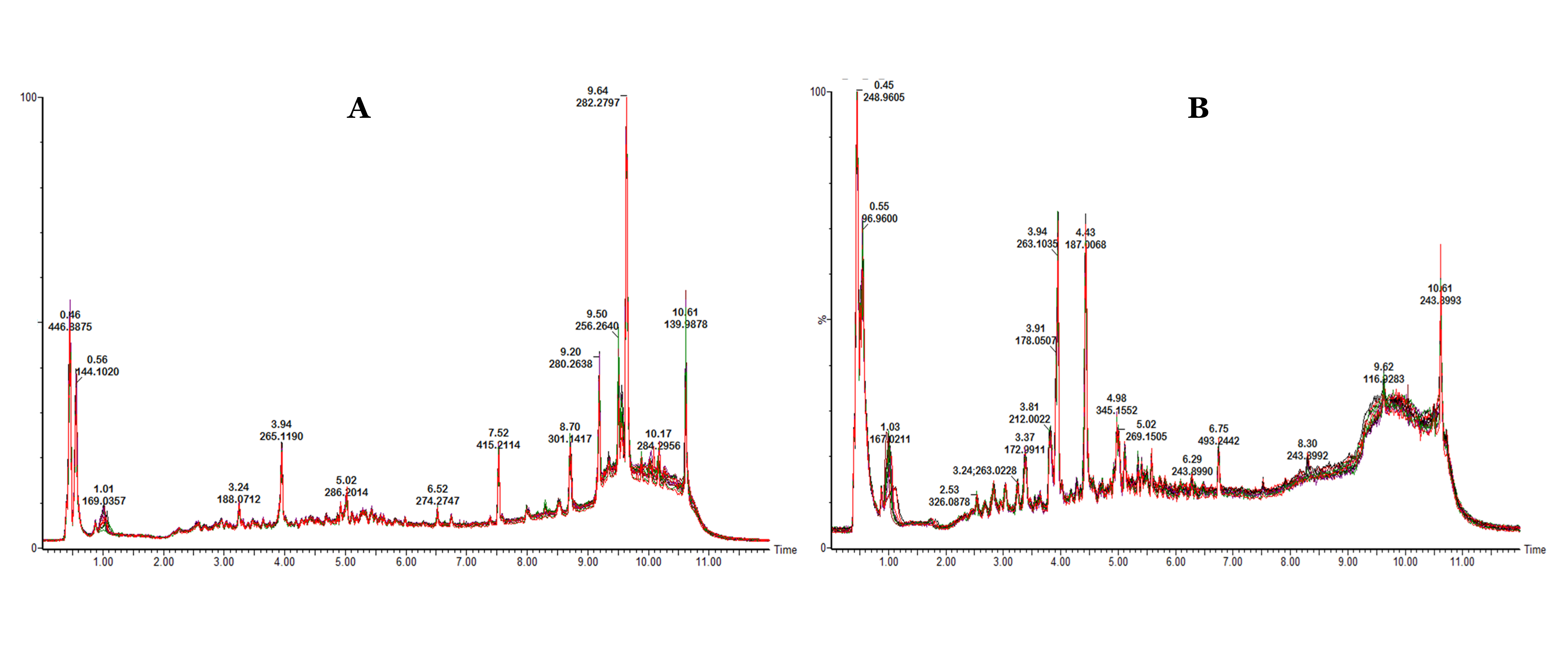

Supplement: Supplementary Figure 1 — Identification of DEPs. The X-axis represents protein difference (log2-transformed fold changes), and the Y-axis the corresponding -log10-transformed P-values. Red dots indicate significantly upregulated proteins, green dots indicate significantly downregulated proteins, and gray dots indicate no significant change. [file Data_Sheet_1.zip › Figure S2.tif]

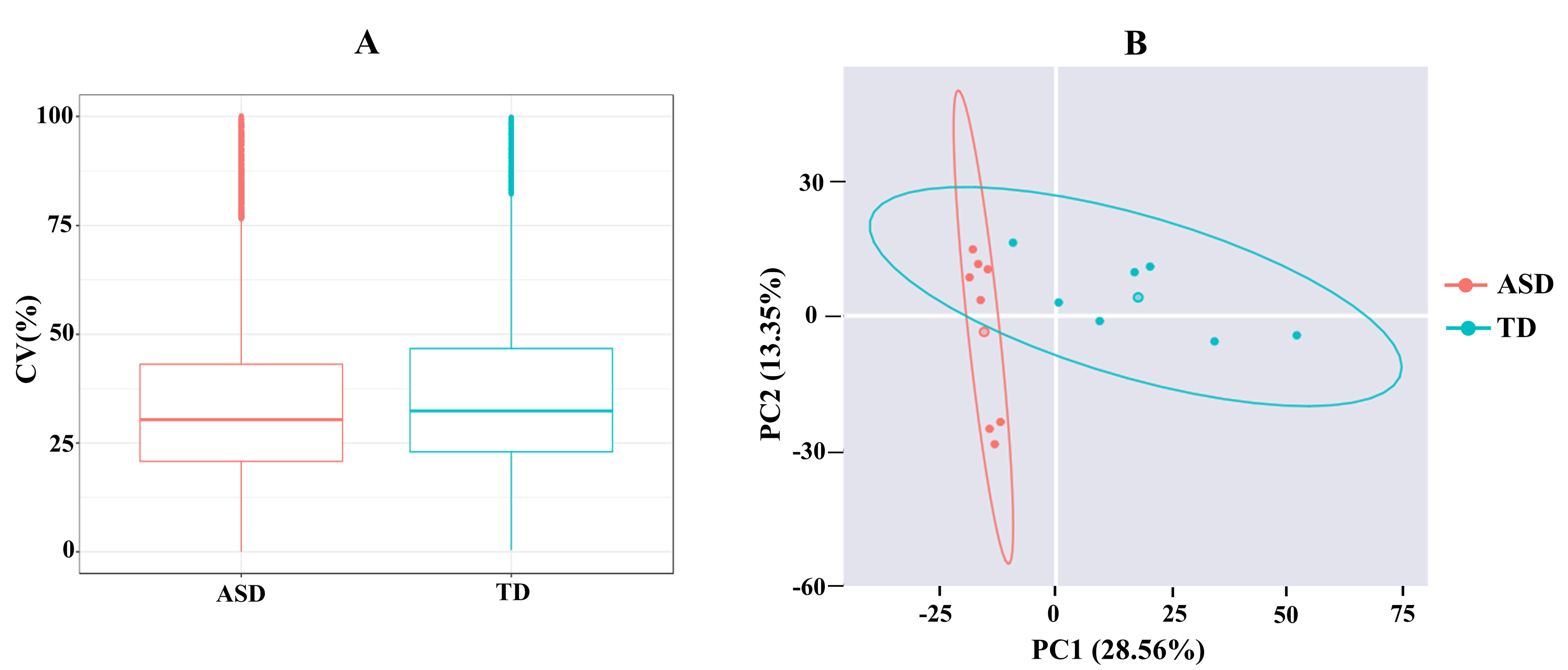

Supplement: Supplementary Figure 1 — Identification of DEPs. The X-axis represents protein difference (log2-transformed fold changes), and the Y-axis the corresponding -log10-transformed P-values. Red dots indicate significantly upregulated proteins, green dots indicate significantly downregulated proteins, and gray dots indicate no significant change. [file Data_Sheet_1.zip › Figure S3.tif]
